# A Genetic Locus within the FMN1/GREM1 Gene Region Interacts with Body Mass Index in Colorectal Cancer Risk

**DOI:** 10.1158/0008-5472.CAN-22-3713

**Published:** 2023-05-30

**Authors:** Elom K. Aglago, Andre Kim, Yi Lin, Conghui Qu, Marina Evangelou, Yu Ren, John Morrison, Demetrius Albanes, Volker Arndt, Elizabeth L. Barry, James W. Baurley, Sonja I. Berndt, Stephanie A. Bien, D. Timothy Bishop, Emmanouil Bouras, Hermann Brenner, Daniel D. Buchanan, Arif Budiarto, Robert Carreras-Torres, Graham Casey, Tjeng Wawan Cenggoro, Andrew T. Chan, Jenny Chang-Claude, Xuechen Chen, David V. Conti, Matthew Devall, Virginia Diez-Obrero, Niki Dimou, David Drew, Jane C. Figueiredo, Steven Gallinger, Graham G. Giles, Stephen B. Gruber, Andrea Gsur, Marc J. Gunter, Heather Hampel, Sophia Harlid, Akihisa Hidaka, Tabitha A. Harrison, Michael Hoffmeister, Jeroen R. Huyghe, Mark A. Jenkins, Kristina Jordahl, Amit D. Joshi, Eric S. Kawaguchi, Temitope O. Keku, Anshul Kundaje, Susanna C. Larsson, Loic Le Marchand, Juan Pablo Lewinger, Li Li, Brigid M. Lynch, Bharuno Mahesworo, Marko Mandic, Mireia Obón-Santacana, Victor Moreno, Neil Murphy, Hongmei Nan, Rami Nassir, Polly A. Newcomb, Shuji Ogino, Jennifer Ose, Rish K. Pai, Julie R. Palmer, Nikos Papadimitriou, Bens Pardamean, Anita R. Peoples, Elizabeth A. Platz, John D. Potter, Ross L. Prentice, Gad Rennert, Edward Ruiz-Narvaez, Lori C. Sakoda, Peter C. Scacheri, Stephanie L. Schmit, Robert E. Schoen, Anna Shcherbina, Martha L. Slattery, Mariana C. Stern, Yu-Ru Su, Catherine M. Tangen, Stephen N. Thibodeau, Duncan C. Thomas, Yu Tian, Cornelia M. Ulrich, Franzel JB van Duijnhoven, Bethany Van Guelpen, Kala Visvanathan, Pavel Vodicka, Jun Wang, Emily White, Alicja Wolk, Michael O. Woods, Anna H. Wu, Natalia Zemlianskaia, Li Hsu, W. James Gauderman, Ulrike Peters, Konstantinos K. Tsilidis, Peter T. Campbell

**Affiliations:** 1 Department of Epidemiology and Biostatistics, Imperial College London, School of Public Health, London, United Kingdom.; 2 Department of Population and Public Health Sciences, Keck School of Medicine, University of Southern California, Los Angeles, California.; 3 Public Health Sciences Division, Fred Hutchinson Cancer Research Center, Seattle, Washington.; 4 Division of Cancer Epidemiology and Genetics, National Cancer Institute, National Institutes of Health, Bethesda, Maryland.; 5 Division of Clinical Epidemiology and Aging Research, German Cancer Research Center (DKFZ), Heidelberg, Germany.; 6 Department of Epidemiology, Geisel School of Medicine at Dartmouth, Hanover, New Hampshire.; 7 Bioinformatics and Data Science Research Center, Bina Nusantara University, Jakarta, Indonesia.; 8 BioRealm LLC, Walnut, California.; 9 Leeds Institute of Cancer and Pathology, University of Leeds, Leeds, United Kingdom.; 10 Department of Hygiene and Epidemiology, University of Ioannina School of Medicine, Ioannina, Greece.; 11 Division of Preventive Oncology, German Cancer Research Center (DKFZ) and National Center for Tumor Diseases (NCT), Heidelberg, Germany.; 12 German Cancer Consortium (DKTK), German Cancer Research Center (DKFZ), Heidelberg, Germany.; 13 Colorectal Oncogenomics Group, Department of Clinical Pathology, The University of Melbourne, Parkville, Victoria, Australia.; 14 University of Melbourne Centre for Cancer Research, Victorian Comprehensive Cancer Centre, Parkville, Victoria, Australia.; 15 Genomic Medicine and Family Cancer Clinic, The Royal Melbourne Hospital, Parkville, Victoria, Australia.; 16 Computer Science Department, School of Computer Science, Bina Nusantara University, Jakarta, Indonesia.; 17 ONCOBELL Program, Bellvitge Biomedical Research Institute (IDIBELL), L'Hospitalet de Llobregat, Barcelona, Spain.; 18 Digestive Diseases and Microbiota Group, Girona Biomedical Research Institute (IDIBGI), Salt, Girona, Spain.; 19 Center for Public Health Genomics, Department of Public Health Sciences, University of Virginia, Charlottesville, Virginia.; 20 Division of Gastroenterology, Massachusetts General Hospital and Harvard Medical School, Boston, Massachusetts.; 21 Channing Division of Network Medicine, Brigham and Women's Hospital and Harvard Medical School, Boston, Massachusetts.; 22 Clinical and Translational Epidemiology Unit, Massachusetts General Hospital and Harvard Medical School, Boston, Massachusetts.; 23 Broad Institute of Harvard and MIT, Cambridge, Massachusetts.; 24 Department of Epidemiology, Harvard T.H. Chan School of Public Health, Harvard University, Boston, Massachusetts.; 25 Department of Immunology and Infectious Diseases, Harvard T.H. Chan School of Public Health, Harvard University, Boston, Massachusetts.; 26 Division of Cancer Epidemiology, German Cancer Research Center (DKFZ), Heidelberg, Germany.; 27 University Medical Centre Hamburg-Eppendorf, University Cancer Centre Hamburg (UCCH), Hamburg, Germany.; 28 Medical Faculty Heidelberg, Heidelberg University, Heidelberg, Germany.; 29 Department of Family Medicine, University of Virginia, Charlottesville, Virginia.; 30 Unit of Biomarkers and Susceptibility (UBS), Oncology Data Analytics Program (ODAP), Catalan Institute of Oncology (ICO), L'Hospitalet del Llobregat, Barcelona, Spain.; 31 Consortium for Biomedical Research in Epidemiology and Public Health (CIBERESP), Madrid, Spain.; 32 Department of Clinical Sciences, Faculty of Medicine, University of Barcelona, Barcelona, Spain.; 33 Novo Nordisk Foundation Center for Basic Metabolic Research, Faculty of Health and Medical Sciences, University of Copenhagen, Denmark.; 34 Nutrition and Metabolism Branch, International Agency for Research on Cancer, World Health Organization, Lyon, France.; 35 Department of Medicine, Samuel Oschin Comprehensive Cancer Institute, Cedars-Sinai Medical Center, Los Angeles, California.; 36 Lunenfeld Tanenbaum Research Institute, Mount Sinai Hospital, University of Toronto, Toronto, Ontario, Canada.; 37 Cancer Epidemiology Division, Cancer Council Victoria, Melbourne, Victoria, Australia.; 38 Centre for Epidemiology and Biostatistics, Melbourne School of Population and Global Health, The University of Melbourne, Melbourne, Australia.; 39 Precision Medicine, School of Clinical Sciences at Monash Health, Monash University, Clayton, Victoria, Australia.; 40 Department of Medical Oncology & Therapeutics Research, City of Hope National Medical Center, Duarte California.; 41 Center for Cancer Research, Medical University of Vienna, Vienna, Austria.; 42 Department of Radiation Sciences, Oncology Unit, Umeå University, Umeå, Sweden.; 43 Center for Gastrointestinal Biology and Disease, University of North Carolina, Chapel Hill, North Carolina.; 44 Department of Genetics, Stanford University, Stanford, California.; 45 Department of Computer Science, Stanford University, Stanford, California.; 46 Institute of Environmental Medicine, Karolinska Institutet, Stockholm, Sweden.; 47 University of Hawaii Cancer Center, Honolulu, Hawaii.; 48 Centre for Epidemiology and Biostatistics, Melbourne School of Population and Global Health, The University of Melbourne, Melbourne, Australia.; 49 Physical Activity Laboratory, Baker Heart and Diabetes Institute, Melbourne, Australia.; 50 Department of Clinical Sciences, Faculty of Medicine, University of Barcelona, Barcelona, Spain.; 51 Department of Epidemiology, Richard M. Fairbanks School of Public Health, Indianapolis, Indiana.; 52 IU Melvin and Bren Simon Cancer Center, Indiana University, Indianapolis, Indiana.; 53 Department of Pathology, School of Medicine, Umm Al-Qura'a University, Mecca, Saudi Arabia.; 54 Department of Epidemiology, University of Washington School of Public Health, Seattle, Washington.; 55 Program in MPE Molecular Pathological Epidemiology, Department of Pathology, Brigham and Women's Hospital, Harvard Medical School, Boston, Massachusetts.; 56 Department of Oncologic Pathology, Dana-Farber Cancer Institute, Boston, Massachusetts.; 57 Huntsman Cancer Institute, Salt Lake City, Utah.; 58 Department of Population Health Sciences, University of Utah, Salt Lake City, Utah.; 59 Department of Laboratory Medicine and Pathology, Mayo Clinic Arizona, Scottsdale, Arizona.; 60 Department of Medicine, Boston University School of Medicine, Slone Epidemiology Center, Boston University, Boston, Massachusetts.; 61 Department of Epidemiology, Johns Hopkins Bloomberg School of Public Health, Baltimore, Maryland.; 62 Research Centre for Hauora and Health, Massey University, Wellington, New Zealand.; 63 Department of Community Medicine and Epidemiology, Lady Davis Carmel Medical Center, Haifa, Israel.; 64 Ruth and Bruce Rappaport Faculty of Medicine, Technion-Israel Institute of Technology, Haifa, Israel.; 65 Clalit National Cancer Control Center, Haifa, Israel.; 66 Department of Nutritional Sciences, University of Michigan School of Public Health, Ann Arbor, Michigan.; 67 Division of Research, Kaiser Permanente Northern California, Oakland, California.; 68 Department of Genetics and Genome Sciences, Case Western Reserve University, Cleveland, Ohio.; 69 Genomic Medicine Institute, Cleveland Clinic, Cleveland, Ohio.; 70 Department of Medicine and Epidemiology, University of Pittsburgh Medical Center, Pittsburgh, Pennsylvania.; 71 Department of Internal Medicine, University of Utah, Salt Lake City, Utah.; 72 SWOG Statistical Center, Fred Hutchinson Cancer Research Center, Seattle, Washington.; 73 Division of Laboratory Genetics, Department of Laboratory Medicine and Pathology, Mayo Clinic, Rochester, Minnesota.; 74 School of Public Health, Capital Medical University, Beijing, China.; 75 Division of Human Nutrition and Health, Wageningen University & Research, Wageningen, the Netherlands.; 76 Wallenberg Centre for Molecular Medicine, Umeå University, Umeå, Sweden.; 77 Department of Molecular Biology of Cancer, Institute of Experimental Medicine of the Czech Academy of Sciences, Prague, Czech Republic.; 78 Institute of Biology and Medical Genetics, First Faculty of Medicine, Charles University, Prague, Czech Republic.; 79 Faculty of Medicine and Biomedical Center in Pilsen, Charles University, Pilsen, Czech Republic.; 80 Memorial University of Newfoundland, Discipline of Genetics, St. John's, Canada.; 81 Department of Biostatistics, University of Washington, Seattle, Washington.; 82 Department of Epidemiology and Population Health, Albert Einstein College of Medicine, Bronx, New York.

## Abstract

Colorectal cancer risk can be impacted by genetic, environmental, and lifestyle factors, including diet and obesity. Gene-environment interactions (G × E) can provide biological insights into the effects of obesity on colorectal cancer risk. Here, we assessed potential genome-wide G × E interactions between body mass index (BMI) and common SNPs for colorectal cancer risk using data from 36,415 colorectal cancer cases and 48,451 controls from three international colorectal cancer consortia (CCFR, CORECT, and GECCO). The G × E tests included the conventional logistic regression using multiplicative terms (one degree of freedom, 1DF test), the two-step EDGE method, and the joint 3DF test, each of which is powerful for detecting G × E interactions under specific conditions. BMI was associated with higher colorectal cancer risk. The two-step approach revealed a statistically significant G×BMI interaction located within the Formin 1/Gremlin 1 (*FMN1/GREM1*) gene region (rs58349661). This SNP was also identified by the 3DF test, with a suggestive statistical significance in the 1DF test. Among participants with the CC genotype of rs58349661, overweight and obesity categories were associated with higher colorectal cancer risk, whereas null associations were observed across BMI categories in those with the TT genotype. Using data from three large international consortia, this study discovered a locus in the *FMN1/GREM1* gene region that interacts with BMI on the association with colorectal cancer risk. Further studies should examine the potential mechanisms through which this locus modifies the etiologic link between obesity and colorectal cancer.

**Significance:**

This gene-environment interaction analysis revealed a genetic locus in FMN1/GREM1 that interacts with body mass index in colorectal cancer risk, suggesting potential implications for precision prevention strategies.

## Introduction

Colorectal cancer is a multifactorial disease that results from many genetic and behavioral/lifestyle risk factors, including diet and obesity (1, 2). General obesity, usually defined in adult populations as a body mass index (BMI) equal to or above 30 kg/m^2^ has been consistently associated with higher risk of colorectal cancer and is estimated to account for 5% to 14% of all colorectal cancer diagnosed cases (3, 4). Mechanistically, obesity affects colorectal cancer carcinogenesis through different pathways, including adipose tissue-associated inflammation, oxidative stress, impairment of lipid metabolism, alterations of the microbiome, and through its causal association with comorbidities such as type 2 diabetes (5). Obesity is a worldwide health issue, mainly attributed to the ubiquitously expanded obesogenic environment (6), with a worldwide prevalence that ranged from less than 5% in the 1970s to over 13% in 2016 (i.e., more than 650 million adults worldwide; ref. 7).

Understanding how inherited germline genetic variation can influence colorectal cancer risk according to obesity status is important for developing better disease risk assessment tools. In recent years, genome-wide association studies (GWAS) have identified over 200 genetic risk variants associated with colorectal cancer development (8–12). Overall, the genetic heritability of colorectal cancer is estimated to be up to 20% (13). Given the complexity of colorectal carcinogenesis, gene-environment interactions (G×E) may be particularly well suited for identifying novel susceptibility loci and biologically plausible interactions that could further elucidate important carcinogenic mechanisms. To date, few studies have comprehensively investigated G×E interactions in the context of BMI and colorectal cancer risk (14–16). Interactions have been observed between BMI and known colorectal cancer GWAS loci on rs4779584 (secretogranin V, *SCG5*; ref. 15) and rs4939827 (*SMAD7*; ref. 14).

Because of the large risks for false discovery and ensuing penalties for multiple comparisons, the discovery of new G×E interactions may be limited by statistical power and sample size requirements (17, 18). In this study, we assessed G×BMI interactions using combined data from three large existing international colorectal cancer consortia using GWAS and BMI data.

## Materials and Methods

### Study participants

Our study sample consisted of individual level genomic and epidemiological data from 34 studies (50% prospective cohort studies) included in the Colorectal Cancer Family Registry (CCFR), Colorectal Cancer Transdisciplinary Study (CORECT), and Genetics and Epidemiology of Colorectal Cancer Consortium (GECCO). Control participants were matched by age, sex, genetically defined ancestry, and enrollment date/trial group, when applicable. Cases were defined as invasive colon or rectal tumors and were confirmed via a combination of sources, including clinical records, oncopathologic reports, state, or provincial cancer registries, and/or death certificates. A small subset of cases included were advanced adenomas (*n* = 4,623, 5.4%) confirmed by sigmoidoscopy or colonoscopy. Each study was approved by the relevant ethics committees or review boards pertaining to their institutions. All participants provided written informed consent during recruitment and all studies were conducted in accordance with ethical guidelines relevant to their geographic location and calendar year of enrollment (e.g., Declaration of Helsinki, CIOMS, Belmont Report, U.S. Common Rule).

### Data harmonization and BMI assessment

Data harmonization consisted of a multistep procedure performed at the GECCO consortium coordinating center at the Fred Hutchinson Cancer Center as previously described (19). In brief, common data elements (CDE) were defined *a priori* for data harmonization. These CDEs represent common variables such as age and sex, or similar variables such as smoking or dietary intake. CDEs ensure that each variable defined is similar and comparable across different studies, hence allowing statistical analysis across a combined dataset. Study questionnaires and data dictionaries were examined, and elements were mapped to these CDEs through an iterative process of communication with data contributors. Definitions, permissible values, and standardized coding were implemented in a single database via SAS (RRID:SCR_008567) and T-SQL. The resulting data were checked for errors and outliers within and between the studies.

Demographic, anthropometric, and lifestyle variables such as sex, age, smoking, and self-reported or measured weight and height were collected via in-person interviews or structured self-administered questionnaires. In the cohort studies, standing height and body weight were ascertained at the time of blood collection or buccal swabs. In case–control studies, standing height was recalled at enrollment for cases and controls, except in REACH, where height was recalled a year prior to the time of interview in cases and controls, and in DACHS, where it was recalled at the time of enrollment in controls and diagnosis in cases. Body weight was recalled 1 to 2 years prior to enrollment of controls or diagnosis of cases in most case–control studies to avoid bias from illness-associated weight loss, except for DACHS and DALS where prediagnostic (in cases) and pre-enrollment (in controls) recall times were 5 to 14 years and 2 to 5 years, respectively. In CRCGEN, weight was recalled at the age of 45 years in cases and controls or 10 years before diagnosis in cases. BMI was calculated as the weight (kg) of each participant divided by the square of the height (m²). We scaled the BMI to reflect a 5 kg/m² increment as our main analytical variable. In addition, for categorical analyses, we used the World Health Organization (WHO) predefined BMI cut-off points for normal weight (18.5 to <25 kg/m²), overweight (≥25.0 to <30 kg/m²), and obesity (≥30 kg/m²). We excluded a small number of participants with a BMI below 18.5 kg/m² (*n* = 677) because of reported nonlinear associations between BMI and colorectal cancer risk at this end of the BMI continuum (20).

### Genotyping and imputation

DNA was extracted from blood or buccal samples. Genotype data were generated from germline DNA on the Affymetrix Axiom (CORECT), UK Biobank Axiom (UK Biobank), Illumina 1M, 1M-Duo, or Omni1 (CCFR), Illumina 300 K, Illumina OmniExpress, Illumina 550K/610 K, or Affymetrix 100K/500 K (GECCO), and Illumina Omni 2.5 in the Molecular Epidemiology of Colorectal Cancer Study (MECC) as detailed elsewhere (8, 10) and summarily presented in Supplementary Table S1. High-density genotype array data were cleaned by applying standard quality control filters at both individual subject and SNP levels. Genotype data for case and control participants were phased and imputed together by an array platform, thus avoiding any potentially differential imputation error between case and control participants.

Participants were excluded based on genotyping call rates (<97%), heterozygosity, duplicates or close propinquity, and inconsistencies between self-reported and genotypic sexes. We limited the analyses to individuals of European ancestry (3,586 participants of non-European descent were excluded) as determined by genetically defined ancestry and principal components (PC) clustering results with 1000 Genomes EUR populations. In terms of markers, we excluded SNPs based on missing call rates (>2 to 5%), departure from Hardy-Weinberg Equilibrium (*P* < 1×10^−4^), and discordant genotype calls within duplicate samples. Genotypes were imputed to the Haplotype Reference Consortium (version r1.1) using the University of Michigan Imputation Server (21). To facilitate data management and analysis, genotypes were converted into a binary format using the BinaryDosage R package (https://cran.r-project.org/web/packages/BinaryDosage). We filtered imputed SNPs based on imputation accuracy of R² > 0.8 and minor allele frequency (MAF) > 1%. Over 7.2 million SNPS were retained after imputation and quality control, among which ∼1 million SNPs were evaluated in our analysis because of the correlation between SNPs. PC analysis for population stratification assessment was performed using PLINK1.9 (RRID:SCR_001757) on 30,000 randomly selected imputed SNPs with MAF and R^2^ over 5% and 0.99, respectively. colorectal cancer molecular subtypes, that is, *BRAF* and *KRAS* mutation status, CpG island methylator phenotype (CIMP), and microsatellite instability (MSI), were analyzed in the subset with available tumor samples (6,565 cases; Supplementary Table S2). *BRAF*, CIMP, *KRAS*, and MSI were determined using specific markers assessed using PCR, sequencing, or IHC.

### Statistical analyses

Logistic regression models were run to examine associations between BMI and colorectal cancer risk in each study, and the results for all the studies were summarized using random-effects meta-analysis methods (Hartung-Knapp) to obtain summary ORs and 95% confidence intervals (CI). We calculated the heterogeneity *P* values using Cochran's Q statistics, while funnel plots identified studies with potential outlying ORs. We fitted the models overall and further stratified them by study design, sex, and tumor site in the colon and rectum. All meta-analyses of BMI main effects were performed using the R package Meta (RRID:SCR_019055; ref. 22).

We performed genome-wide interaction scans using the R package G×EScanR (23), which implements several interaction testing methods. Our analyses included conventional logistic regression with multiplicative interaction terms (1 degree of freedom test, 1DF), two-step EDGE method (17), and 3DF analyses (24), as detailed in the Supplementary methods. Compared with the 1DF, the joint 3DF test has higher power to detect G×E interactions when they exist, while accommodating the main effect of each variant on risk of colorectal cancer (D|G) and the effect of the environment on colorectal cancer risk (E|G) associations (24). The two-step method reduces the burden of multiple testing by preserving the statistical power, mainly through the initial filtering step that incorporates the D|G and E|G associations. We presented the 1DF analysis and the two-step method, followed by the 3DF test. The rationale for presenting the results in this order is that both the 1DF and two-step method test directly for G×E interactions, the first as a classic test and the latter as a generally more powerful test. The 3DF analysis is presented last because it tests interaction indirectly and is particularly powerful in the presence of association between G and D or between G and E induced by interaction.

To identify novel loci, we filtered out previously known GWAS hits and SNPs identified in linkage disequilibrium (LD) with them in the 1000 Genomes EUR dataset. All models were adjusted for study, sex, age at diagnosis or enrollment, and the first three PCs of genetic ancestry to account for population substructure. For SNPs that reached statistical significance (*P* = 5.0×10^−8^/3 = 1.67×10^−8^, to account for three main tests) or showed a trend towards that threshold (*P* < 5×10^−6^), we performed stratified G×E analyses by sex, tumor site (proximal colon, distal colon, rectum), and study design. We also added smoking status (never/ever), an established risk factor for colorectal cancer, to the multivariate models. We did not adjust for other lifestyle or dietary variables associated with colorectal cancer, such as alcohol consumption, physical inactivity, intake of red and processed meats, or calcium, because they do not interact with the SNPs and their inclusion in previous genetic analyses in our consortium (25) did not materially change the associations with colorectal cancer.

We calculated ORs stratified by BMI categories (normal, overweight, obese) and genotype to examine the patterns of stratum-specific associations. These models were fitted using imputation posterior probabilities and visualized with a plot of predicted log-odds versus genotype by BMI category. As the positive association between obesity and colorectal cancer risk was consistently stronger in males than in females, we also explored the G×BMI interactions separately by sex. We used logistic regression (cases/controls; mutated/nonmutated) and nominal polytomous logistic regression (mutated/nonmutated/controls) to explore the interactions between the significant SNPs and BMI on colorectal tumor molecular subtypes, that is, *BRAF* and *KRAS* mutation status, CIMP, and MSI.

Functional annotations were examined for the significant findings, as described in the Supplementary methods. In brief, the magnitude of the association, the extent of the association explained by LD, as well as chromosomal position and neighboring SNPs and genes were investigated. Regional plots were generated using the command line version (Standalone) of LocusZoom v1.3 (26). The putative functional role of the SNPs with significant interactions and those in LD (*r*^2^ > 0.2) at 500 kb flanking regions were explored relative to their potential contribution to regulate gene expression by their physical location in regions of chromatin accessibility or histone modifications (variant enhancer loci). Genes where expression in colon tissue samples were regulated by functional SNPs (*P* values below 5×10^−4^) were identified using the colon transverse tissue samples from GTEx v8 dataset, and the colon transcriptome explorer (CoTrEx 2.0; https://barcuvaseq.org/cotrex, accessed January 2023) of the University of Barcelona and University of Virginia genotyping and RNA sequencing (BarcUVA-Seq) project dataset (27). This dataset is comprised of 445 epithelium-enriched healthy colon biopsies from ascending, transverse, and descending colon. We retrieved for each significant SNP, the genes with available expression and located within one million base pairs. The gene expressions were fit in a gene only model, and subsequentially in a model including the interaction between the continuous gene expression and BMI, and a model with the gene expression in categories.

All statistical analyses were performed using R version 3.6.0. (RRID:SCR_001905, Foundation for Statistical Computing).

### Data and code availability

The datasets and code supporting the current study have not been deposited in a public repository because they are part of an international consortium but are available from the corresponding author upon request.

## Results

A total of 36,415 colorectal cancer cases (17,139 females) and 48,451 control (23,717 females) participants were included in this analysis. Mean age (SD) of the participants was 63.8 (10.4) and 62.4 (9.7) years in case and control participants, respectively (Table 1).

**Table 1.** Selected characteristics of the participants.

	Cases (*n* = 36,415)	Controls (*n* = 48,451)
Females, %	47.1	49.0
Age, yrs, mean ± SD	63.8 ± 10.4	62.4 ± 9.7
**Anthropometry, mean ± SD**		
Height, cm	169 ± 9.6	169 ± 9.6
BMI, kg/m²	27.5 ± 4.8	27.1 ± 4.6
Socio-demographic and lifestyle, %		
Education (highest completed)		
Less than high school	26.9	21.7
High school/GED	18.8	14.7
Some college	22.5	24.7
College/graduate school	27.3	32.2
Smoking, ever		
No	44.5	49.0
Yes	53.1	49.5
**Medical information, %**		
Type 2 diabetes (ever diagnosed)		
No	81.7	85.0
Yes	11.8	8.4
Any postmenopausal HRT use		
No	20.3	21.8
Yes	11.1	13.3
Regular aspirin or NSAID use		
No	53.9	51.2
Yes	27.9	34.2
**Dietary intake, Mean ± SD**		
Energy, kcal/day	1,950 ± 760	1,890 ± 717
Calcium, mg/day	790 ± 442	809 ± 440
Folate, mcg/day	373 ± 217	389 ± 222
Fiber, g/day	19.5 ± 9.8	19.8 ± 9.7
Red meat, servings/day	0.7 ± 0.6	0.6 ± 0.6
Processed meat, servings/day	0.4 ± 0.4	0.3 ± 0.4
Fruit, servings/day	2.0 ± 1.7	2.2 ± 1.8
Vegetable, servings/day	2.9 ± 2.5	2.6 ± 2.5

Note: Frequencies may not add up to 100 due to missing values.

Abbreviations: GED, general educational development; HRT, hormone replacement therapy; NSAID, nonsteroidal anti-inflammatory drugs.

**Figure 1.**
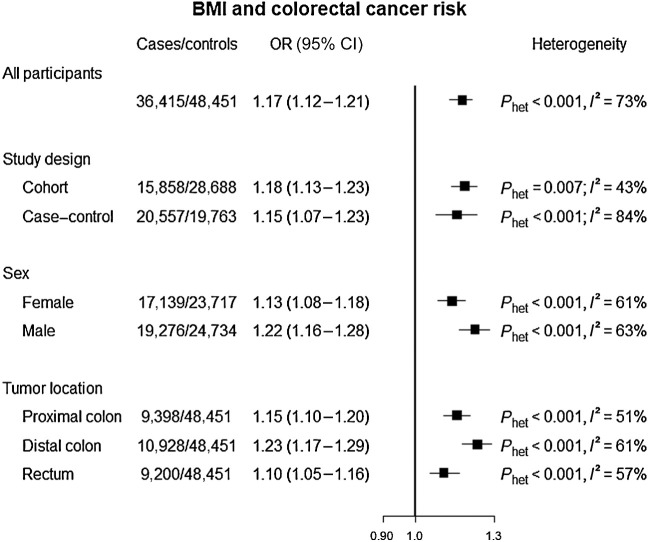
OR and 95% CI for colorectal cancer risk associated with BMI (per 5 kg/m^2^ increment). The OR and 95% CIs were calculated for individual participating studies and then meta-analyzed.

In a meta-analysis of all the participating studies, each 5 kg/m^2^ increase in BMI was associated with 17% higher risk of colorectal cancer (OR = 1.17; 95% CI, 1.12–1.21; Fig. 1). The positive association was consistent across study designs, sex, and tumor anatomical subsites, although the estimated BMI effect was higher in males (OR = 1.22; 95% CI, 1.16–1.28) compared with females (OR = 1.13; 95% CI, 1.08–1.18; *P*_interaction_ = 4.47×10^−8^) and in the distal colon (OR = 1.23; 95% CI, 1.17–1.29) compared with the proximal colon (OR = 1.15; 95% CI, 1.10–1.20; *P*_interaction_ = 0.003) or the rectum (OR = 1.10; 95% CI, 1.05–1.16; *P*_interaction_ = 9.0×10^−8^).

Table 2 summarizes the most prominent SNP×BMI interactions. The quantile-quantile (Q-Q) plot for G×BMI interaction for the 1DF analysis did not reveal residual stratification in the study population (Supplementary Fig. S1). In the 1DF exploration using multiplicative interaction terms in logistic regression models, we did not observe any genome-wide statistically significant loci interacting with BMI on colorectal cancer risk (Supplementary Fig. S2). Nine SNPs with *P* values below 5×10^−6^ in the 1DF are presented in Supplementary Table S3.

**Table 2.** Summary of G × BMI analyses using 1DF, two-step, and 3DF analyses.

										*P* values for each method			
Method	G×E significant	Position^a^	Gene	chr	A1	A2	MAF	*P*_(D|G)_ ^b^	*P*_(E|G)_ ^c^	*P*_1DF_	*P*_step 1 EDGE_ ^d^	*P*_step 2 EDGE_ ^e^	*P*_3DF_
1DF	*None*												
Two-step EDGE	15 SNPs (all in LD)^f^												
rs58349661	–	33122966	*FMN1*	15	C	T	0.21	4.3 × 10^−7^	3.7 × 10^−10^	5.0 × 10^−6^	2.22 × 10^−6^	4.97 × 10^−6^	3.7 × 10^−10^
3DF	12 SNPs (E|G driven)^g^												

Abbreviations: DF, degrees of freedom; EDGE, elastic data-driven genetic encoding.

^a^Position based on NCBI Build37.

^b^Association between genetic variant and colorectal cancer.

^c^Association between genetic variant and BMI.

^d^Two-step EDGE filtering *P* value.

^e^Two-step EDGE second step *P* value.

^f^Five SNPs, all located on *FMN1* and LD (presented in Supplementary Tables). The SNP with the lowest *P* value was rs58349661.

^g^Twelve SNPs were significant for G×BMI in the 3DF analyses (presented in Supplementary Tables). None of these SNPs had a marginal G effect, except for rs58349661 (previously observed in the two-step approach) and rs7313400. The latter was not significant in the 1DF analysis (*P*_1DF_ = 0.07).

In the two-step EDGE approach, we observed a statistically significant interaction between variants located in the formin 1/Gremlin 1 (*FMN1/GREM1*) gene region and BMI in colorectal cancer risk (Supplementary Fig. S3). The lead SNP with the lowest *P* value was rs58349661 (*P*_step 2_ = 4.97×10^−6^ < *P*_threshold_ = 4.3×10^−5^), which was in high LD with surrounding SNPs showing similar significant interactions (Supplementary Table S4). The 3DF test also identified this locus interacting with BMI on colorectal cancer risk (rs58349661 *P*_3DF_ = 3.68×10^−10^; Supplementary Table S3). Consistent with these findings, we observed suggestive findings for SNPs in this locus in the 1DF test (rs58349661 *P*_1DF_ = 4.97×10^−6^) as well as strong association with colorectal cancer (rs58349661 *P*_(D|G)_ = 4.3×10^−7^) and BMI (rs58349661 *P*_(E|G)_ = 3.7×10^−10^).

**Figure 2.**
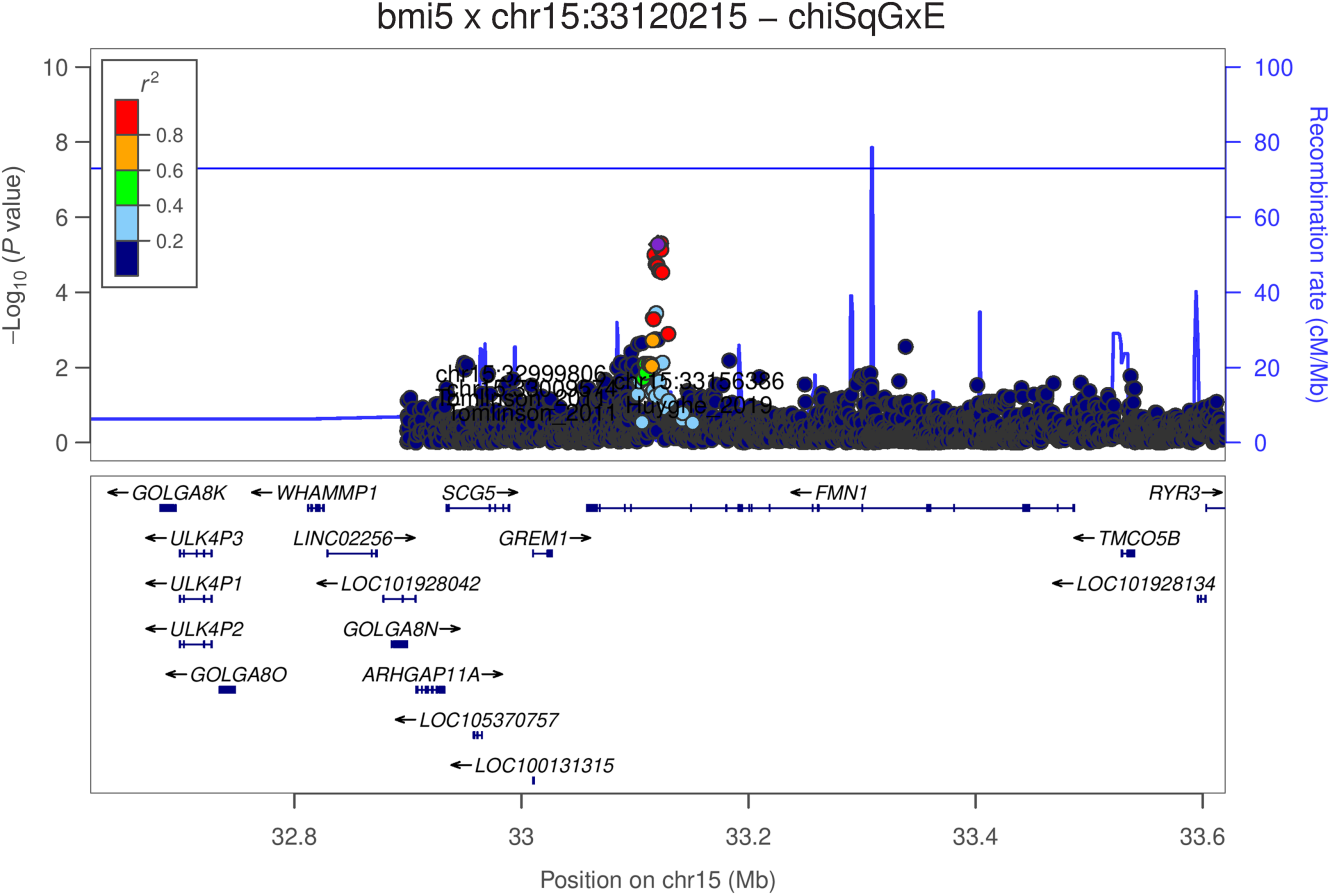
LocusZoom plots for the interaction between rs58349661 (rs1975678 used as perfect LD proxy) and colorectal cancer. The genomic position is shown on the *x*-axis whereas the *y*-axis reports the −log_10_ of the *P* value of the interaction with BMI. Purple dot, rs1975678. The colors of the SNPs are based on their correlation with rs1975678. Previously known GWAS variants, with their references, are also included: rs17816465, rs1919364, rs12708491.

This locus is within the *FMN1/GREM1* gene region with *SCG5* and has transmembrane and coiled-coil domains 5 B *(TMCO5B)* as neighboring genes (LocusZoom plot for rs1975678, Fig. 2). While investigating the relationship between rs58349661 and previously studied genetic variants, we found that this locus is close to, but not in strong LD (R² = 0.002 to 0.162) with genetic variants previously associated with colorectal cancer in *FMN1* (rs16959063, rs17816465), *GREM1* (rs10318, rs1919364), and *SCG5* (rs16969681, rs4779584; refs. 8, 28, 29). None of these variants showed a significant interaction with BMI after correction for multiple testing (Fig. 2).

**Figure 3.**
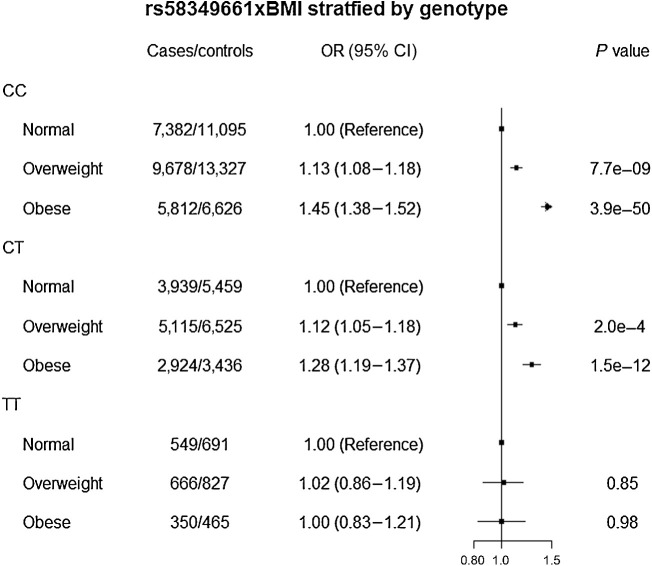
BMI in relation to colorectal cancer risk, stratified by genotype. Using normal BMI as the reference, risk for colorectal cancer was estimated for overweight and obesity across the genotypes of rs58349661.

Among participants with CC and CT genotypes of rs58349661, overweight (OR_CC_ = 1.13; 95% CI, 1.08–1.18; OR_CT_ = 1.12; 95% CI, 1.05–1.18) and obesity (OR_CC_ = 1.45; 95% CI, 1.38–1.52; OR_CT_ = 1.28; 95% CI, 1.19–1.37) were associated with higher colorectal cancer risk, whereas null associations were observed across BMI categories in those with the TT genotype (OR_TT_ overweight = 1.02; 95% CI, 0.86–1.19; OR_TT_ obesity = 1.00; 95% CI, 0.83–1.21; Fig. 3). Similarly, analyses stratified by BMI showed that the CT and TT genotypes were associated with a higher colorectal cancer risk in participants with normal BMI or overweight, but the risk was null in obese participants (Supplementary Table S5). Analyses of rs58349661×BMI stratified by sex, study design, and tumor anatomical location showed similar results as in the whole study population (Supplementary Table S6). The interaction between the T allele of rs58349661 and BMI did not differ across tumor molecular subtypes (all *P* values comparing mutated vs. nonmutated for *BRAF* mutation, *KRAS* mutation, CIMP, and MSI were > 0.05; Supplementary Fig. S4).

We did not observe that rs58349661 or correlated SNPs (e.g., rs1975678, which is in perfect LD with rs58349661, R^2^ = 1, Supplementary Table S4) were associated with gene expression. rs58349661 was not associated with gene expression in colon tissues in the GTEx v8 data. Nevertheless, rs58349661 (or variants in LD) were expressed in other tissues, especially in cultured fibroblasts. These SNPs reside in a region with high H3K27ac activity in normal colonic epithelium, but this activity is lost in tumor cells (Supplementary Fig. S5). Genes near variant rs58349661 identified via the BarcUVa dataset were *AC123768.3*, Rho GTPase activating protein 11A (*ARHGAP11A*), apoptosis and caspase activation inhibitor (*AVEN*), cholinergic receptor nicotinic alpha 7 subunit (*CHRNA7*), *FMN1*, and ryanodine receptor 3 (*RYR3*), of which, a significant interaction with BMI was observed for *AVEN* (*P*_interaction_ = 0.04) and *CHRNA7* (*P*_interaction_ = 0.04; Supplementary Table S7).

## Discussion

In this study from three large international colorectal cancer consortia, we discovered a new locus located within the *FMN1/GREM1* gene region (rs58349661) that interacts with BMI on the association with colorectal cancer risk. Analyses stratified by genotypes of rs58349661 showed that obesity and overweight were associated with a higher risk of colorectal cancer in those with the CC genotype but not in those with the TT genotype, which showed consistently null associations across BMI categories.

Our novel G×BMI finding at the *FMN1/GREM1* locus is close to a region with multiple known GWAS loci for colorectal cancer risk. Among the ∼200 SNPs associated with colorectal cancer in GWAS, an increasing number of independent loci have been located within the broader *SCG5-GREM1-FMN1* region (rs17816465, rs10318, rs1919364, rs16969681, and rs4779584; refs. 8, 30, 31). The lead SNP (rs58349661), as well as other correlated SNPs in our interaction analysis, has not been previously observed in GWAS analyses for colorectal cancer risk. Moreover, none of these genetic variants were in strong LD with previously known loci, suggesting that this interaction locus was independent of previously known loci. Our finding of a higher colorectal cancer risk associated with obesity and the CC genotype, and null associations with the TT genotype, suggests that T and C alleles may participate in specific mechanisms that influence the effect of obesity on colorectal cancer risk.

The mechanisms by which obesity affects colorectal cancer risk are not fully understood, but several plausible hypotheses have been proposed, including the colonic microenvironment and related systemic inflammation, oxidative stress, and major changes in other metabolic activities, including hyperinsulinemia and diabetes mellitus (32). At the histologic and cellular levels, obesity originates from uncontrolled hyperplasia and/or hypertrophy of the adipocytes. GWAS studies on adiposity traits, including BMI, have not reported any associations with SNPs within *FMN1* gene (33–37). This is consistent with gene expression studies that did not report the major activity of *FMN1* in matured adipocytes (38). In contrast to adipocytes, *FMN1* is expressed in the colon and rectal mucosal cells (38). *FMN1* is known to interact with alpha-catenin in the production of adherens junctions and polymerization of actin monomers (39). Hence, this phenomenon is mainly observed during the formation of epithelial sheets in adipocyte stem cells when linear actin cables and microtubules are formed at adherens junctions (40). This may explain why *FMN1* activity is not observed in fully developed adipocytes. *FMN1* has been reported as an epigenomic region associated with early childhood adiposity, a period in which adipose tissue is still in development (41).

Although very little is known about *FMN1* and colorectal cancer, there are extensive data on formins, more generally, their role in developmental biology, WNT signaling, and their association with colorectal cancer. Humans have 15 Formin genes including *FMN1* (38). These genes are defined by a ∼400 amino acid formin homology-2 (FH2) domain, which is the actin nucleation apparatus responsible for eukaryotic actin filament assembly and elongation (42, 43). FH2 influences actin dynamics and is common in all Formins across multiple eukaryotic species (44). Formins were originally identified at the mouse limb deformity locus (45) and *FMN1* particularly is central to one of the human syndactyly disorders (46), another one of which involves a novel splicing mutation in *APC* that results in an ∼80% reduction in the wildtype transcript (47). Several Formins (including *DAAM1* and *DAAM2*) are key components of canonical WNT signaling in cancer development (48, 49), thus they are involved in *APC* mutation and colon polyp formation. A variety of Formins but not *FMN1*, have been implicated in invasion and metastasis of colorectal cancer (38). Additional studies are necessary to fully understand the possible contribution of *FMN1* to colorectal tumor development according to obesity status.

In a previous G×BMI analysis, we observed a locus located within *SMAD7* that interacts with BMI on colorectal cancer risk. Our current analytical strategy excluded *SMAD7* and other known GWAS loci for colorectal cancer and BMI. TGFβ in cellular proliferation and inflammation has been proposed as a mechanism of action through which *SMAD7* interacts with colorectal cancer risk (50). In contrast to the tumor-promoting profile of *SMAD7* in obesity, *FMN1* does not have a clear relationship with inflammation. It is possible that *FMN1* acts in tandem with neighboring genes, such as *GREM1* and *SCG5* because the catena *SCG5-GREM1-FMN1* constitutes a hotspot for several colorectal cancer–related SNPs. Animal studies have shown that *GREM1* may be activated by a cis-regulatory region within *FMN1* (51). Given that the SNPs interacting with BMI are located within the enhancer region, as indicated by high H3K27ac activity, it might also be possible that these SNPs impact *GREM1*. Moreover, *GREM1* has been associated with cancer fibroblasts in colonic and rectal smooth muscles and colonic crypt bases, and may participate in colorectal tumorigenesis through bone morphogenetic protein (BMP) signaling (52). Mutations in *GREM1* are associated with the development of hereditary mixed polyposis syndrome (HMPS), a rare condition associated with an increased development of colon polyps and higher colorectal cancer risk, often beginning in childhood. Overexpression of *GREM1*, as observed in HMPS, acts as a BMP antagonist, thus allowing the cells to conserve stem properties and develop into neoplasia (53). Experimental studies using animal and human models have shown that increased expression of *GREM1* can induce epithelial dedifferentiation through BMP signaling and initiate gut cells neoplasia (54, 55). Future studies should specifically aim to unveil the potential mechanisms by which this SNP interacts with obesity on the association with colorectal cancer risk.

The main strength of our study was the sample size, the largest ever to have examined G×BMI associations. The use of several complementary statistical approaches was also a strength because it allowed clear characterization of a specific locus within *FMN1/GREM1* that was consistently captured in the two-step and 3DF analyses, with a suggestive association indicated by the 1DF test. One limitation of our study is that it included only participants of European ancestry; hence, our findings may not be generalizable to other populations. Notably, the T allele of rs58349661 is common in other populations, with frequencies of 0.186, 0.518, 0.210, 0.307, 0.216 in African, East Asian, European, South Asian, and American populations, respectively. The A allele (rs58349661 is triallelic) is rare in all populations (frequency in Asian, European, and American populations ≈0), except in the African population where it has a frequency of 0.059. Another limitation is that a recall time of 1 to 2 years prediagnosis in some (but not all) case–control studies may not be enough to rule out weight loss due to the tumor. Such weight loss may also have affected the BMI at recruitment in colorectal cancer cases occurring during the early years of follow-up in cohort studies.

In conclusion, we identified a new locus in the *FMN1/GREM1* gene region that interacts with BMI on the association with colorectal cancer risk. This locus has not been previously described in relation to obesity or colorectal cancer, and additional investigation is required to elucidate the potential mechanisms by which it may modify the detrimental effects of obesity in promoting colorectal carcinogenesis.

## Supplementary Material

Supplementary Data supplementary materials
