# Supplementary material for: A Genetic Locus within the FMN1/GREM1 Gene Region Interacts with Body Mass Index in Colorectal Cancer Risk
Source: Cancer Res. 2023 May 30;83(15):2572–83. doi: 10.1158/0008-5472.CAN-22-3713 (PMC10391330; doi:10.1158/0008-5472.CAN-22-3713)
Supplement: Supplementary Data — supplementary materials [file can-22-3713_supplementary_data_suppsm.docx]

**Supplementary materials**

**A genetic locus within the FMN1/GREM1 gene region interacts with body mass index in colorectal cancer risk**

Elom K Aglago, Andre Kim, Yi Lin, Conghui Qu, Marina Evangelou, Ren Yu, John Morrison, Demetrius Albanes, Volker Arndt, Elizabeth L Barry, James W Baurley, Sonja I Berndt, Stephanie A Bien, D Timothy Bishop, Emmanouil Bouras, Hermann Brenner, Daniel D Buchanan, Arif Budiarto, Robert Carreras-Torres, Graham Casey, Tjeng Wawan Cenggoro, Andrew T Chan, Jenny Chang-Claude, Xuechen Chen, David V Conti, Matthew Devall, Virginia Diez-Obrero, Niki Dimou, David Drew, Jane C Figueiredo, Steven Gallinger, Graham G. Giles, Stephen B Gruber, Andrea Gsur, Marc J Gunter, Heather Hampel, Sophia Harlid, Akihisa Hidaka, Tabitha A Harrison, Michael Hoffmeister, Jeroen R Huyghe, Mark A Jenkins, Kristina Jordahl, Amit D Joshi, Eric S. Kawaguchi, Temitope O Keku, Anshul Kundaje, Susanna C Larsson, Loic Le Marchand, Juan Pablo Lewinger, Li Li, Brigid M Lynch, Bharuno Mahesworo, Marko Mandic, Mireia Obón-Santacana, Victor Moreno, Neil Murphy, Hongmei Nan, Rami Nassir, Polly A Newcomb, Shuji Ogino, Jennifer Ose, Rish K Pai, Julie R Palmer, Nikos Papadimitriou, Bens Pardamean, Anita R Peoples, Elizabeth A Platz, John D Potter, Ross L Prentice, Gad Rennert, Edward Ruiz-Narvaez, Lori C Sakoda, Peter C Scacheri, Stephanie L Schmit, Robert E Schoen, Anna Shcherbina, Martha L Slattery, Mariana C Stern, Yu-Ru Su, Catherine M Tangen, Stephen N Thibodeau, Duncan C Thomas, Yu Tian, Cornelia M Ulrich, Franzel JB van Duijnhoven, Bethany Van Guelpen, Kala Visvanathan, Pavel Vodicka, Jun Wang, Emily White, Alicja Wolk, Michael O Woods, Anna H Wu, Natalia Zemlianskaia, Li Hsu, W. James Gauderman, Ulrike Peters, Kostas Tsilidis, Peter T Campbell

| **Page** | **Title** |
| --- | --- |
| 2 | **Figure S1:** Q-Q for the GxBMI association with colorectal cancer |
| 3 | **Figure S2:** Manhattan plots for the GxBMI interactions associations |
| 4 | **Figure S3:** Two-step EDGE test plot of top SNPs for gene and body mass index interaction for colorectal risk cancer |
| 5 | **Figure S4:** Interactions between rs58349661 and BMI on colorectal cancer risk by molecular subtypes |
| 6 | **Figure S5:** Functional annotation plot for rs58349661 (*FMN1*) and surrounding genes |
| 7-12 | **Table S1:** Description and count of participants in the participation studies |
| 13 | **Table S2:** Sample size analyzed for molecular subtypes and by study center |
| 14-15 | **Table S3:** Prominent SNPs found using 1DF and 3DF tests |
| 16 | **Table S4:** GxBMI results for the significant SNPs in the two-step method |
| 17 | **Table S5:** Association between rs58349661 and colorectal cancer stratified by BMI categories |
| 18 | **Table S6:** Odds ratios and 95% confidence intervals for colorectal cancer association with rs58349661 and body mass index stratified by study type, sex, and tumour site. |
| 19-22 | **Table S7:** Gene expression by BMI interactions, for genes of the BarcUVa dataset near rs58349661 |
| 23-25 | **Supplementary methods** |
| 26-31 | **Funding/Acknowledgements** |

**A**

**
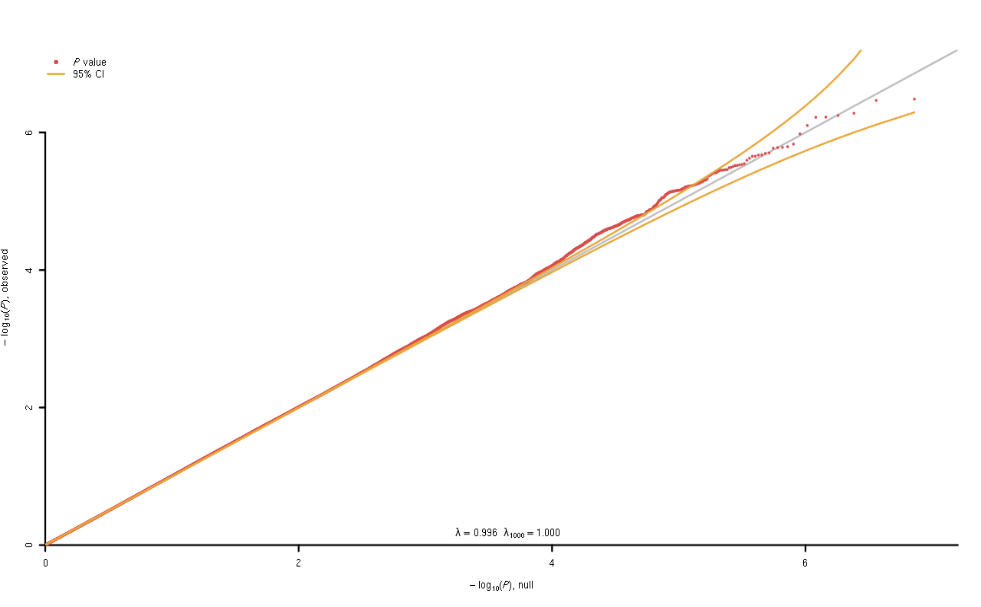
**

**B**

**
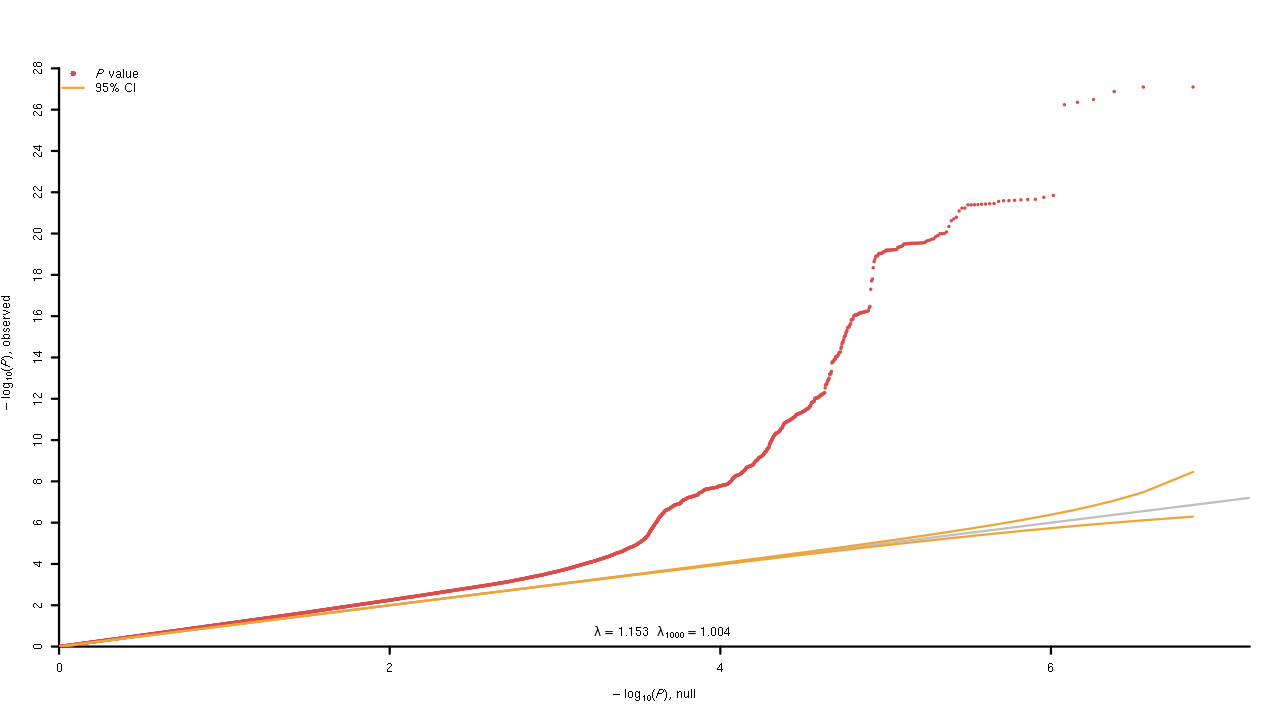
**

**Figure S1:** Q-Q for the GxBMI association with colorectal cancer

A-1DF, B- 3DF

**A**

**
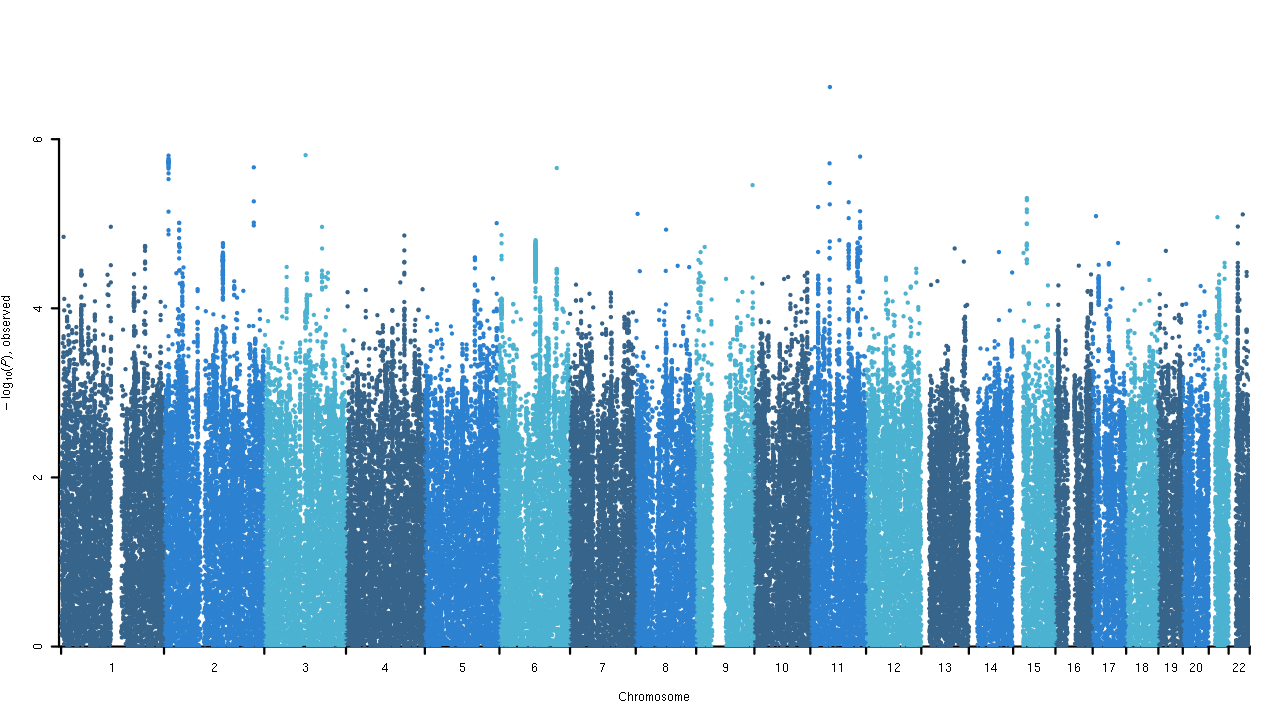
**

**B**

**
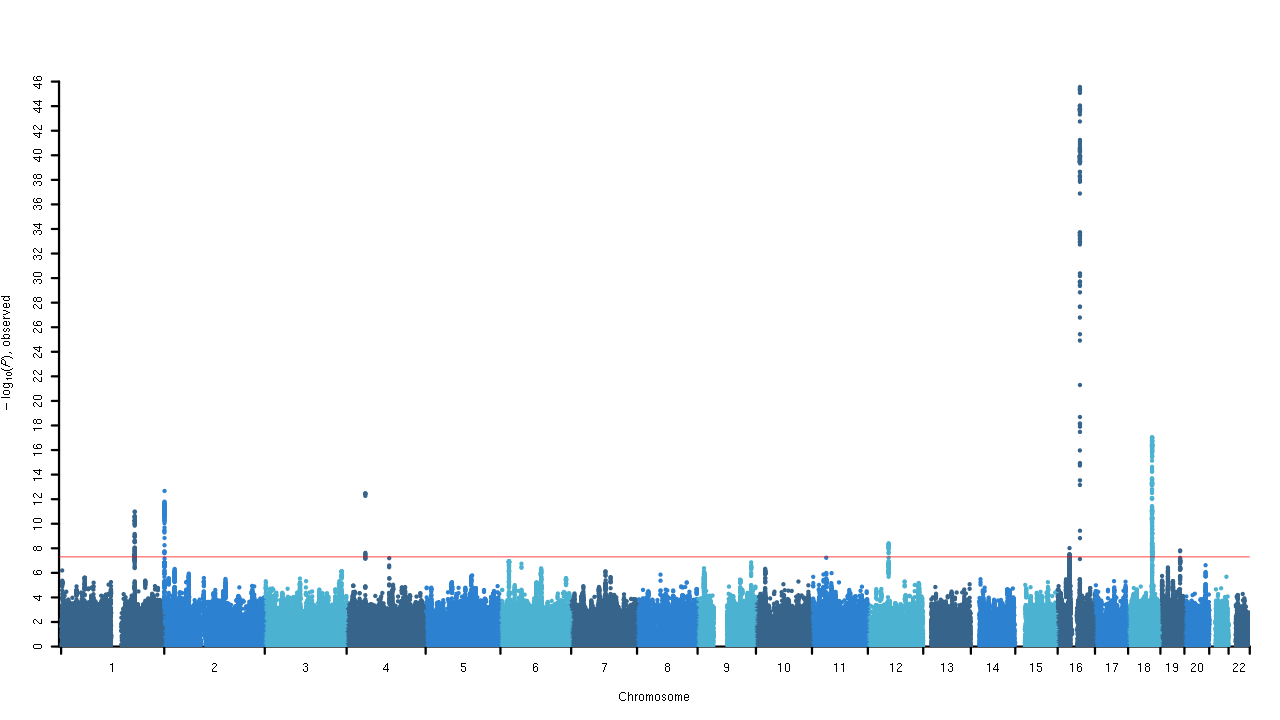
**

**Figure S2:** Manhattan plots for the GxBMI interactions associations

A-1DF, B- 3DF

**
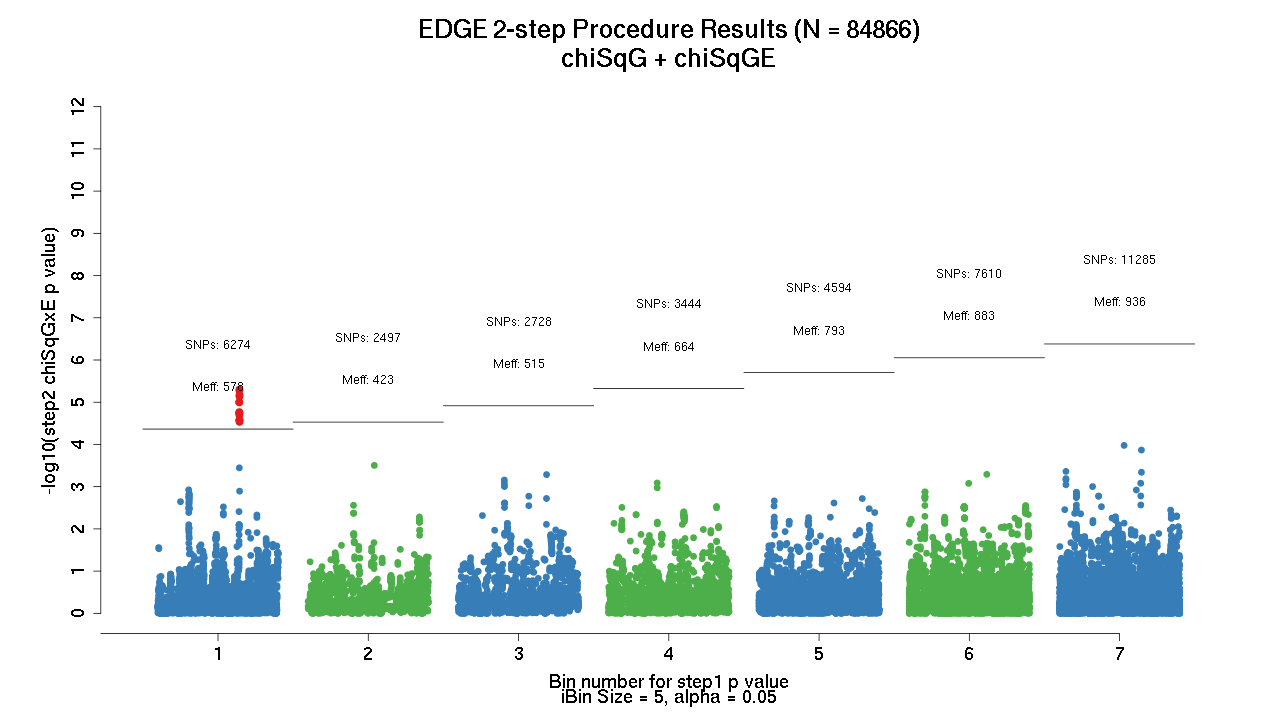
**

**Figure S3:** Two-step EDGE test plot of top SNPs for gene and body mass index interaction for colorectal risk cancer

In blue and green are displayed the SNPs by bin. The number of SNPs per bin is included above each bin, as is the effective number of independent tests (Meff) based on principal components analysis of the SNPs within each bin (see Methods). The SNPs above the cutpoints (red dots) achieved genome-wide significance for the two-step procedure.


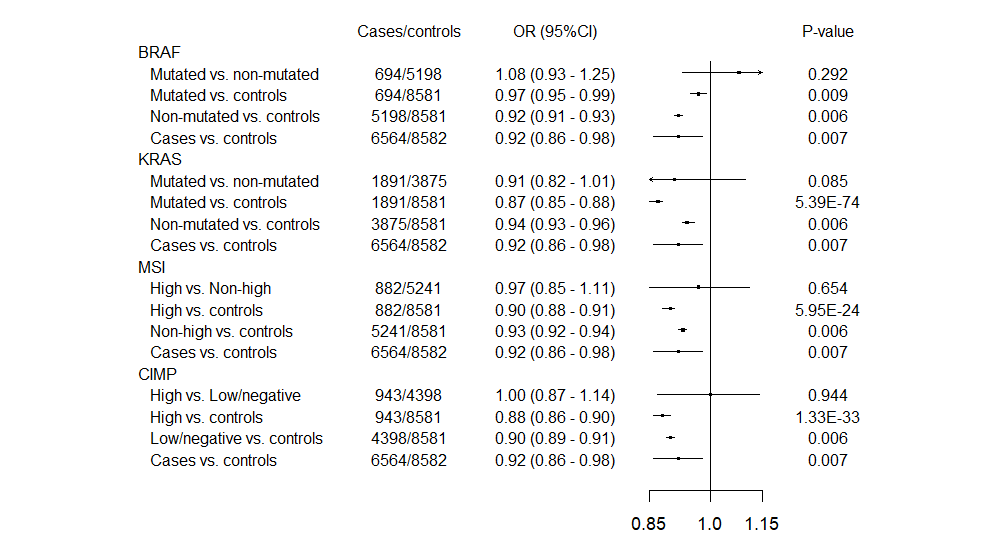


**Figure S4:** Interactions between rs58349661 and BMI on colorectal cancer risk by molecular subtypes

**
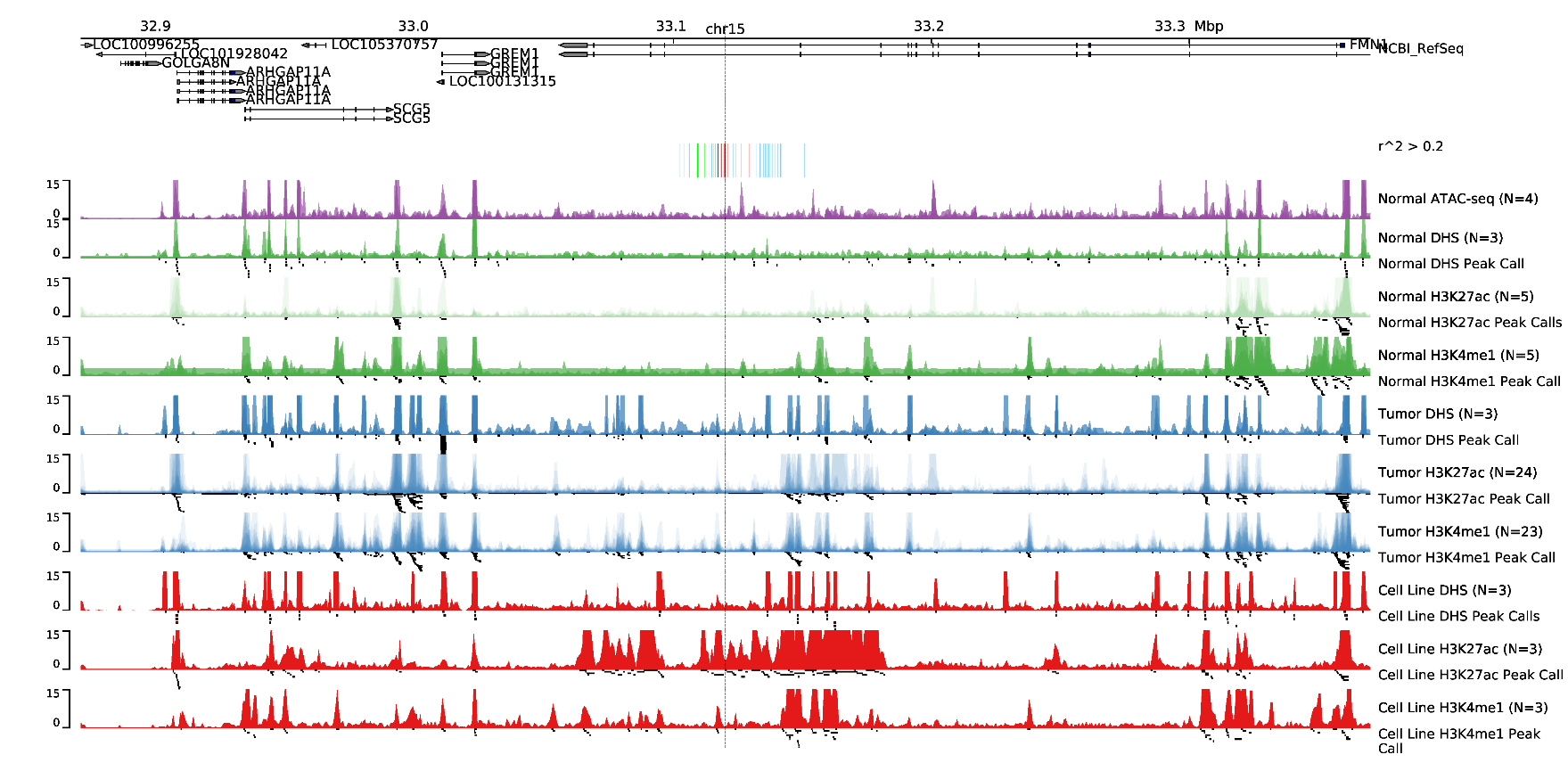
**

**Figure S5:** Functional annotation plot for rs58349661 (*FMN1*) and surrounding genes

**Table S1:** Description and count of participants in the participation studies

| **Study Acronym** | **Study Name** | **Study Design** | **Country** | **Genotyping Platform** | **N Cases** | **N Controls** | **Mean BMI±SD in cases** | **Mean BMI±SD in controls** |
| --- | --- | --- | --- | --- | --- | --- | --- | --- |
| ATBC | Alpha-Tocopherol, Beta Carotene Cancer Prevention Study | cohort | Finland | Illumina Oncoarray | 152 | 32 | 26.8 ± 3.8 | 26.3 ± 4.9 |
| CCFR_1 | Colon Cancer Family Registry | case-control | USA, Canada, Australia | Illumina 1M, 1M duo | 988 | 957 | 27.6 ± 5.3 | 26.8 ± 5.1 |
| CCFR_3 | Colon Cancer Family Registry | case-control | USA, Canada, Australia | Affymetrix Axiom | 836 | 627 | 27 ± 5.1 | 26.7 ± 4.7 |
| CCFR_4 | Colon Cancer Family Registry | case-control | USA, Canada, Australia | Illumina Oncoarray | 1141 | 640 | 27.9 ± 5.8 | 26.5 ± 4.9 |
| CLUEII | Campaign against Cancer and Heart Disease II | cohort | USA | Illumina Oncoarray+ custom iSelect | 264 | 244 | 26.5 ± 4.2 | 26 ± 3.7 |
| Colo2&3 | Hawai’i Colorectal Cancer Studies 2&3 | case-series | USA | Illumina 300K | 74 | 106 | 27 ± 6 | 25.4 ± 4.7 |
| CORSA_2 | Colorectal Cancer Study of Austria | case-control | Austria | Affymetrix Axiom | 1097 | 1159 | 28.4 ± 4.7 | 28.1 ± 4.8 |
| CORSA_1 | Colorectal Cancer Study of Austria | case-control | Austria | Illumina Oncoarray+ custom iSelect | 944 | 693 | 27.9 ± 4.6 | 28.3 ± 4.5 |
| CPSII_1 | American Cancer Society Cancer Prevention Study II nested case-control study | cohort | USA | Affymetrix Axiom | 494 | 490 | 26.9 ± 4.6 | 26 ± 3.8 |
| CPSII_2 | American Cancer Society Cancer Prevention Study II nested case-control study | cohort | USA | Illumina Oncoarray+ custom iSelect | 307 | 319 | 26.4 ± 4.3 | 25.9 ± 3.8 |
| CRCGEN | Colorectal Cancer Genetics & Genomics, Spanish study | case-control | Spain | Illumina Oncoarray | 821 | 958 | 26.5 ± 4 | 26.9 ± 4.2 |
| CzechCCS | Czech Republic CCS | case-control | Czech Republic | Illumina Oncoarray+ custom iSelect | 1257 | 1478 | 27.3 ± 4.4 | 26.4 ± 4.1 |
| DACHS_1 | Darmkrebs: Chancen der Verhütung durch Screening | case-control | Germany | Illumina 300K | 1608 | 1654 | 27.1 ± 4.1 | 26.4 ± 3.7 |
| DACHS_2 | Darmkrebs: Chancen der Verhütung durch Screening | case-control | Germany | Illumina OmniExpress | 644 | 480 | 27.2 ± 3.9 | 26.2 ± 3.7 |
| DACHS_3 | Darmkrebs: Chancen der Verhütung durch Screening Study | case-control | Germany | Illumina OmniExpressExome | 1190 | 609 | 27.4 ± 4.2 | 26.3 ± 4.2 |
| DALS_2 | Diet, Activity and Lifestyle Study | case-control | USA | Illumina 550K, 610K | 397 | 454 | 27.8 ± 5.1 | 26.6 ± 4.6 |
| DALS_1 | Diet, Activity and Lifestyle Study | case-control | USA | Illumina 300K | 688 | 695 | 27.9 ± 5.2 | 26.5 ± 4.3 |
| EDRN | Early Detection Research Network | case cohort | USA | Illumina Oncoarray+ custom iSelect | 231 | 289 | 29.7 ± 6.6 | 26.9 ± 5 |
| EPIC | European Prospective Investigation into Cancer and Nutrition | cohort | Europe | Illumina OmniExpressExome | 2008 | 2311 | 27 ± 4.3 | 26.3 ± 3.8 |
| ESTHER_VERDI | Epidemiologische Studie zu Chancen der Verhütung, Früherkennung und optimierten Therapie chronischer Erkrankungen in der älteren Bevölkerung; Verlauf der diagnotischen Abklärung bei Krebspatienten | case-control | Germany | Illumina Oncoarray | 295 | 428 | 26.6 ± 3.9 | 27.5 ± 3.7 |
| HawaiiCCS_AD | Hawaii Adenoma Study | case-control | USA | Illumina Oncoarray+ custom iSelect | 80 | 525 | 27.6 ± 5.3 | 26.7 ± 4.7 |
| HPFS_3_AD | Health Professionals Follow-Up Study | cohort | USA | Illumina OmniExpress | 301 | 280 | 25.9 ± 3.1 | 25.7 ± 3.1 |
| HPFS_4 | Health Professionals Follow-Up Study | cohort | USA | Illumina OmniExpress | 183 | 195 | 26.6 ± 3.8 | 25.7 ± 3.3 |
| HPFS_1_2 | Health Professionals Follow-Up Study | cohort | USA | Illumina OmniExpressExome | 189 | 238 | 26.1 ± 3.1 | 25.3 ± 2.9 |
| HPFS_5_AD | Health Professionals Follow-Up Study | cohort | USA | Illumina Oncoarray+ custom iSelect | 153 | 103 | 27.3 ± 3.8 | 25.9 ± 3.1 |
| Kentucky | Kentucky Case-Control Study | case-control | USA | Affymetrix Axiom | 862 | 1027 | 29.2 ± 6.1 | 28.1 ± 5.6 |
| LCCS | Leeds Colorectal Cancer Study | case-control | UK | Illumina Oncoarray+ custom iSelect | 1347 | 664 | 26.4 ± 4.4 | 26.6 ± 4.7 |
| MCCS_1 | Melbourne Collaborative Cohort Study | cohort | Australia | Affymetrix Axiom | 534 | 458 | 27.5 ± 4 | 26.9 ± 3.9 |
| MCCS_2 | Melbourne Collaborative Cohort Study | cohort | Australia | Affymetrix Axiom | 218 | 212 | 27.7 ± 4.6 | 27.3 ± 4.1 |
| MEC_1 | Multiethnic Cohort Study | cohort | USA | Illumina 300K | 317 | 336 | 27 ± 5 | 26.1 ± 4.3 |
| MEC_2 | Multiethnic Cohort Study | cohort | USA | Illumina 300K | 63 | 88 | 27.7 ± 5.8 | 25.7 ± 4.1 |
| MECC_1 | Molecular Epidemiology of Colorectal Cancer study | case-control | Israel | Illumina Omni 2.5 | 432 | 462 | 27.2 ± 4.3 | 27 ± 4.3 |
| MECC_2 | Molecular Epidemiology of Colorectal Cancer study | case-control | Israel | Affymetrix Axiom | 1017 | 975 | 27.4 ± 4.6 | 26.8 ± 4.2 |
| MECC_3 | Molecular Epidemiology of Colorectal Cancer study | case-control | Israel | Illumina OncorayIllumina Infinium OncoArra | 3265 | 2618 | 27.9 ± 4.8 | 27.4 ± 4.7 |
| NCCCSI | North Carolina Colon Cancer Study, I | case-control | USA | Illumina Oncoarray+ custom iSelect | 240 | 456 | 27.8 ± 5.7 | 27.8 ± 5.4 |
| NCCCSII | North Carolina Colon Cancer Study, II | case-control | USA | Illumina Oncoarray+ custom iSelect | 555 | 650 | 29.4 ± 6.3 | 28.1 ± 5.4 |
| NFCCR_2 | Newfoundland Case-Control Study 2 | case-control | Canada | Affymetrix Axiom | 183 | 460 | 28.9 ± 5 | 27.4 ± 4.4 |
| NHS_1_2 | Nurses’ Health Study | cohort | USA | Illumina OmniExpress | 354 | 740 | 25.5 ± 4.5 | 25.7 ± 4.3 |
| NHS_3_AD | Nurses’ Health Study | cohort | USA | Illumina OmniExpress | 482 | 402 | 25.8 ± 4.4 | 25.2 ± 4.5 |
| NHS_4 | Nurses’ Health Study | cohort | USA | Illumina OmniExpressExome | 285 | 284 | 27 ± 5.6 | 26.2 ± 5.2 |
| NHS_5_AD | Nurses’ Health Study | cohort | USA | Illumina Oncoarray+ custom iSelect | 245 | 213 | 27 ± 5.5 | 26.1 ± 4.3 |
| PHS | Physicians’ Health Study | cohort | USA | Illumina OmniExpress | 373 | 386 | 25.4 ± 2.7 | 24.8 ± 2.7 |
| PLCO_1_Rematch | Prostate, Lung, Colorectal, and Ovarian Cancer Screening Trial | cohort | USA | Illumina 300/240S & 610K | 523 | 318 | 27.9 ± 5.1 | 27.3 ± 4.7 |
| PLCO_2 | Prostate, Lung, Colorectal, and Ovarian Cancer Screening Trial | cohort | USA | Illumina 300K | 456 | 357 | 27.5 ± 4.4 | 27.2 ± 4.1 |
| PLCO_3 | Prostate, Lung, Colorectal, and Ovarian Cancer Screening Trial | cohort | USA | Illumina 300K | 730 | 3340 | 28 ± 4.8 | 27.2 ± 4.7 |
| PLCO_4_AD | Prostate, Lung, Colorectal, and Ovarian Cancer Screening Trial | cohort | USA | Illumina Oncoarray+ custom iSelect | 1086 | 918 | 28.1 ± 4.8 | 27.5 ± 4.8 |
| PPS3 | Aspirin/Folate Polyp Prevention Study | clinical trial | USA | Illumina OncorayIllumina Infinium OncoArra | 63 | 451 | 27.4 ± 3.4 | 27.3 ± 4.8 |
| PPS4 | Vitamin D/Calcium Polyp Prevention Study | clinical trial | USA | Illumina OncorayIllumina Infinium OncoArra | 143 | 910 | 30 ± 5.6 | 28.7 ± 5.3 |
| REACH_AD | Screening Markers for Colorectal Disease study and Colonoscopy and Health study | case-control | USA | Illumina 300K | 78 | 308 | 28.4 ± 4.6 | 26.9 ± 4.7 |
| SELECT | Selenium and Vitamin E Prevention Trial | clinical trial | USA | Illumina Oncoarray+ custom iSelect | 261 | 268 | 28.9 ± 4.3 | 28.5 ± 4.4 |
| SMC_COSM | Swedish Mammography Cohort and Cohort of Swedish Men | cohort | Sweden | Illumina Oncoarray | 506 | 823 | 25.7 ± 3.4 | 25.4 ± 3.5 |
| SMS_AD | Screening Markers for Colorectal Cancer Study (advanced adenomas) | case-control | USA | Illumina Oncoarray+ custom iSelect | 40 | 125 | 27.2 ± 4.7 | 27.8 ± 5.5 |
| UKB_1 | UK Biobank | cohort | UK | UK Biobank Axiom | 2897 | 11536 | 28 ± 4.6 | 27.6 ± 4.5 |
| USC_HRT_CRC | Los Angeles County Cancer Surveillance Program | case-control | USA | Illumina Oncoarray | 287 | 391 | 27.3 ± 6.1 | 26.6 ± 5.8 |
| VITAL | VITamins And Lifestyle | cohort | USA | Illumina 300K | 254 | 265 | 28.4 ± 5.6 | 27 ± 4.5 |
| WHI_1 | Women’s Health Initiative Study | cohort | USA | Illumina 550K, 550Kduo, 610K | 447 | 505 | 28 ± 5.6 | 26.7 ± 5 |
| WHI_2 | Women’s Health Initiative Study | cohort | USA | Illumina 300K | 972 | 987 | 28.5 ± 5.6 | 28.2 ± 5.6 |
| WHI_3 | Women’s Health Initiative Study | cohort | USA | Illumina Oncoarray+ custom iSelect | 554 | 554 | 28 ± 5.7 | 27.4 ± 5.1 |

Abbreviations: SD, standard deviations

**Table S2:** Sample size analyzed for molecular subtypes and by study center

| Study | Controls | Cases | *BRAF* | | *KRAS* | | CIMP | | MSI | |
| --- | --- | --- | --- | --- | --- | --- | --- | --- | --- | --- |
|  |  |  | Mutated | Non-mutated | Mutated | Non-mutated | High | Low/negative | High | Non-high |
| CCFR_1 | 944 | 983 | 68 | 892 | 285 | 525 | 56 | 673 | 5 | 954 |
| CCFR_3 | 608 | 557 | 106 | 389 | 152 | 329 | 69 | 283 | 162 | 354 |
| CCFR_4 | 625 | 864 | 111 | 703 | 241 | 556 | 112 | 431 | 186 | 595 |
| CPSII_1 | 183 | 189 | 34 | 135 | 56 | 97 | 31 | 143 | 38 | 126 |
| CPSII_2 | 129 | 108 | 8 | 43 | 14 | 23 | 27 | 75 | 11 | 38 |
| DACHS_1 | 1654 | 1285 | 77 | 1050 | 343 | 783 | 161 | 1107 | 111 | 1080 |
| DACHS_2 | 480 | 491 | 45 | 446 | 173 | 318 | 110 | 381 | 57 | 363 |
| DALS_1 | 694 | 527 | 42 | 398 | 148 | 339 | 117 | 330 | 78 | 442 |
| DALS_2 | 454 | 304 | 33 | 210 | 98 | 184 | 74 | 168 | 56 | 246 |
| EDRN | 289 | 170 | 12 | 63 | 28 | 44 | 0 | 0 | 25 | 138 |
| EPIC | 381 | 143 | 33 | 95 | 30 | 100 | 66 | 63 | 22 | 111 |
| HPFS_1_2 | 249 | 121 | 3 | 116 | 45 | 73 | 7 | 87 | 7 | 109 |
| HPFS_4 | 195 | 93 | 9 | 82 | 41 | 50 | 9 | 74 | 13 | 76 |
| MCCS_1 | 458 | 394 | 50 | 316 | 105 | 262 | 39 | 331 | 55 | 338 |
| MCCS_2 | 212 | 83 | 11 | 66 | 26 | 51 | 9 | 66 | 7 | 76 |
| NHS_1_2 | 741 | 148 | 29 | 114 | 63 | 81 | 31 | 111 | 24 | 118 |
| NHS_4 | 285 | 105 | 23 | 80 | 43 | 60 | 25 | 75 | 25 | 77 |

**Table S3**: Prominent SNPs found using 1DF and 3DF tests

| GxE significant | Position^1^ | Gene | Chr | a1 | a2 | a1 frequency | Type | P_(D\|G)_^2^ | P_(E\|G)_^3^ | P_1DF_ | P_3DF_ |
| --- | --- | --- | --- | --- | --- | --- | --- | --- | --- | --- | --- |
| **1DF (None, below P<5x10^-6^)** |  |  |  |  |  |  |  |  |  |  |  |
| rs140025272 | 47328213 | *MADD* | 11 | C | T | 0.0348 | intron | 0.63 | 1.04E-06 | 2.42E-07 | 1.04E-06 |
| rs1228183 | 100037644 | *TBC1D23* | 3 | A | G | 0.3141 | intron | 0.45 | 4.76E-06 | 1.54E-06 | 4.76E-06 |
| rs55771723 | 10961902 | *PDIA6* | 2 | C | T | 0.2972 | intron | 0.12 | 3.12E-06 | 1.56E-06 | 3.12E-06 |
| rs7940234 | 120531547 | *GRIK4* | 11 | C | G | 0.661 | intron | 0.35 | 1.34E-05 | 1.61E-06 | 1.34E-05 |
| rs72912138 | 46574459 | *AMBRA1* | 11 | A | T | 0.0517 | intron | 0.48 | 2.10E-05 | 1.93E-06 | 2.10E-05 |
| rs13409123 | 217410559 | *RPL37A* | 2 | A | G | 0.0249 | intron | 0.10 | 1.27E-05 | 2.15E-06 | 1.27E-05 |
| rs1928277 | 138766021 | *NHSL1* | 6 | C | T | 0.0596 | intron | 0.86 | 4.80E-05 | 2.19E-06 | 4.80E-05 |
| rs62572818 | 136601110 | *SARDH* | 9 | G | C | 0.0596 | intron | 0.23 | 3.18E-05 | 3.49E-06 | 3.18E-05 |
| rs58349661 | 33122966 | *FMN1* | 15 | C | T | 0.2097 | intron | 4.32E-07 | 3.68E-10 | 4.97E-06 | 3.68E-10 |
|  |  |  |  |  |  |  |  |  |  |  |  |
| **two-step method** |  |  |  |  |  |  |  |  |  |  |  |
| rs58349661^4^ | 33122966 | *FMN1* | 15 | C | T | 0.2097 | intron | 4.32E-07 | 3.68E-10 | 4.97E-06 | 3.68E-10 |
|  |  |  |  |  |  |  |  |  |  |  |  |
| **3DF** |  |  |  |  |  |  |  |  |  |  |  |
| rs58349661^4^ | 33122966 | *FMN1* | 15 | C | T | 0.2097 | intron | 4.32E-07 | 3.68E-10 | 4.97E-06 | 3.68E-10 |
| rs1421085 | 53800954 | *FTO* | 16 | C | T | 0.4324 | intron | 0.99 | 1.82E-48 | 0.40 | 2.77E-46 |
| rs17175518 | 57850583 | *RP11-795H16.2* | 18 | A | C | 0.2396 | intergenic | 0.12 | 3.68E-19 | 0.89 | 9.24E-18 |
| rs2867113 | 651365 | *TMEM18* | 2 | A | G | 0.174 | intergenic | 0.10 | 2.46E-14 | 0.26 | 2.16E-13 |
| rs12507026 | 45181334 | *RP11-362I1.1* | 4 | T | A | 0.4195 | intergenic | 0.87 | 5.52E-15 | 0.90 | 3.40E-13 |
| rs62106258 | 417167 | *AC105393.2* | 2 | C | T | 0.0437 | upstream | 0.27 | 6.08E-14 | 0.72 | 1.82E-12 |
| rs543874 | 177889480 | *SEC16B* | 1 | A | G | 0.8131 | downstream | 0.09 | 7.94E-13 | 0.99 | 1.03E-11 |
| rs111526888 | 57971625 | *RP11-396N11.1* | 18 | A | G | 0.7565 | downstream | 0.07 | 5.99E-11 | 1.00 | 5.42E-10 |
| rs7313400 | 50204089 | *NCKAP5L* | 12 | A | G | 0.3618 | intron | 5.42E-03 | 2.36E-08 | 0.07 | 3.85E-09 |
| rs77901086 | 58083923 | *MC4R* | 18 | A | C | 0.994 | intergenic | 0.06 | 1.06E-09 | 0.81 | 7.35E-09 |
| rs35626515 | 28649651 | *NPIPB8* | 16 | C | A | 0.6491 | 5_prime_UTR | 0.36 | 5.63E-10 | 0.33 | 9.55E-09 |
| rs28590228 | 47581242 | *ZC3H4* | 19 | C | T | 0.331 | intron | 0.12 | 5.73E-09 | 0.08 | 1.49E-08 |

^1^Position is based on NCBI Build37

^2^Association between genetic variant and colorectal cancer

^3^Association between genetic variant and BMI

^4^rs58349661 was previously observed and presented in the two-step method

**Table S4:** GxBMI results for the significant SNPs in the two-step method

| Rs ID | Position | Gene | chr | a1 | a2 | type | Bin^1^ | P-value step 1 | P-value step 2 | R² LD^2^ | D' LD^3^ |
| --- | --- | --- | --- | --- | --- | --- | --- | --- | --- | --- | --- |
| rs58349661 | 33122966 | *FMN1* | 15 | C | T | intron | 1 | 2.22E-06 | 4.97E-06 | - | - |
| rs1975678 | 33120215 | *FMN1* | 15 | T | C | intron | 1 | 3.76E-06 | 5.27E-06 | 1 | 1 |
| rs16959250 | 33123010 | *FMN1* | 15 | A | G | intron | 1 | 2.67E-06 | 6.77E-06 | 1 | 1 |
| rs59044286 | 33123143 | *FMN1* | 15 | C | T | intron | 1 | 2.84E-06 | 7.30E-06 | 1 | 0.994 |
| rs921510 | 33117302 | *FMN1* | 15 | C | A | intron | 1 | 3.08E-06 | 9.93E-06 | 0.97 | 1 |
| rs921512 | 33117248 | *FMN1* | 15 | T | C | intron | 1 | 2.97E-06 | 1.01E-05 | 0.97 | 0.994 |
| rs56064734 | 33118742 | *FMN1* | 15 | C | T | intron | 1 | 7.51E-07 | 1.71E-05 | 0.894 | 1 |
| rs8039697 | 33119313 | *FMN1* | 15 | C | T | intron | 1 | 7.11E-07 | 1.79E-05 | 0.894 | 1 |
| rs16959198 | 33118275 | *FMN1* | 15 | C | T | intron | 1 | 6.92E-07 | 1.80E-05 | 0.894 | 1 |
| rs58476108 | 33119777 | *FMN1* | 15 | C | T | intron | 1 | 7.21E-07 | 1.83E-05 | 0.894 | 1 |
| rs59119474 | 33120033 | *FMN1* | 15 | A | C | intron | 1 | 6.72E-07 | 2.01E-05 | 0.894 | 1 |
| rs725807 | 33122379 | *FMN1* | 15 | A | G | intron | 1 | 2.84E-07 | 2.65E-05 | 0.894 | 1 |
| rs74011843 | 33121207 | *FMN1* | 15 | A | G | intron | 1 | 3.43E-07 | 2.65E-05 | 0.888 | 1 |
| rs16959223 | 33121339 | *FMN1* | 15 | A | T | intron | 1 | 3.24E-07 | 2.69E-05 | 0.894 | 1 |
| rs58586003 | 33123979 | *FMN1* | 15 | A | G | intron | 1 | 2.37E-07 | 2.93E-05 | 0.888 | 1 |

^1^SNPs were partitioned in the first bin if they showed P-value < 5x10^-6^ in the first step

²LD R² of the SNPs using the most prominent SNP (rs58349661) as the reference

^3^LD D' of the SNPs using the most prominent SNP (rs58349661) as the reference

**Table S5**: Association between rs58349661 and colorectal cancer stratified by BMI categories

|  | CC | | |  | CT | | |  | TT | | |
| --- | --- | --- | --- | --- | --- | --- | --- | --- | --- | --- | --- |
|  | Cases/controls | OR (95%CI) | P-value |  | Cases/controls | OR (95%CI) | P-value |  | Cases/controls | OR (95%CI) | P-value |
| BMI and genotype with common reference |  |  |  |  |  |  |  |  |  |  |  |
| Normal | 7382/11095 | 1.00 (Reference) | - |  | 3939/5459 | 1.12 (1.06 to 1.18) | 4.3x10^-5^ |  | 549/691 | 1.26 (1.12 to 1.43) | 0.0002 |
| Overweight | 9678/13327 | 1.13 (1.08 to 1.18) | 7.7x10^-9^ |  | 5115/6525 | 1.25 (1.19 to 1.31) | 4.0x10^-18^ |  | 666/827 | 1.28 (1.14 to 1.43) | 1.70 x10^-5^ |
| Obese | 5812/6626 | 1.45 (1.38 to 1.52) | 3.9x10^-50^ |  | 2924/3436 | 1.43 (1.34 to 1.52) | 2.9x10^-30^ |  | 350/465 | 1.26 (1.09 to 1.47) | 0.0022 |

**Table S6**: Odds ratios and 95% confidence intervals for colorectal cancer association with rs58349661 and body mass index stratified by study type, sex, and tumour site.

|  | Cases/controls | T allele | BMI (per 5 unit increment) | T alleleBMI | P for interaction |
| --- | --- | --- | --- | --- | --- |
| All participants | 36415/48451 | 1.50 (1.35- 1.65) | 1.18 (1.16 - 1.20) | 0.94 (0.91 - 0.97) | 4.97E-06 |
| Study design |  |  |  |  |  |
| Cohort | 15858/28688 | 1.58 (1.37 - 1.80) | 1.18 (1.15 - 1.21) | 0.93 (0.89 - 0.97) | 4.08E-04 |
| Case-control | 20557/19763 | 1.43 (1.22 - 1.63) | 1.18 (1.15 - 1.20) | 0.95 (0.91 - 0.98) | 3.38E-03 |
|  |  |  |  |  |  |
| Sex |  |  |  |  |  |
| Female | 17139/23717 | 1.52 (1.33 - 1.71) | 1.14 (1.11 - 1.16) | 0.94 (0.90 - 0.97) | 3.72E-04 |
| Male | 19276/24734 | 1.46 (1.22 - 1.69) | 1.24 (1.21 - 1.27) | 0.94 (0.90 - 0.99) | 7.97E-03 |
|  |  |  |  |  |  |
| Tumour location |  |  |  |  |  |
| Proximal colon | 9398/48451 | 1.55 (1.32 - 1.78) | 1.24 (1.21 - 1.27) | 0.93 (0.89 - 0.97) | 7.39E-04 |
| Distal colon | 10928/48451 | 1.53 (1.30 - 1.75) | 1.18 (1.16 - 1.21) | 0.94 (0.90 - 0.98) | 1.06E-03 |
| Rectum | 9200/48451 | 1.56 (1.31 - 1.81) | 1.12 (1.09 - 1.16) | 0.94 (0.89 - 0.98) | 3.80E-03 |

**Table S7**: Gene expression by BMI interactions, for genes of the BarcUVa dataset near rs58349661

| **Genes and expressions** | Estimate | SE | P-value | n |  |  | Estimate | SE | P-value |
| --- | --- | --- | --- | --- | --- | --- | --- | --- | --- |
| **Using standardised values of gene expression** |  |  |  |  |  | **Using gene expression categories** | |  |  |
| ***AC123768.3*** | | | |  |  |  |  |  |  |
| **Expression Only** |  |  |  |  |  | **Expression Only** |  |  |  |
| BMI | 0.1379 | 0.0079 | 5.73E-68 |  |  | BMI | 0.1379 | 0.0079 | 5.92E-68 |
| AC123768.3.std | -0.0025 | 0.0073 | 0.7341 |  |  | AC123768.3.ter | -0.0209 | 0.0254 | 0.4096 |
| **Interaction Model** |  |  |  |  |  | **Interaction Model** |  |  |  |
| BMI | 0.1380 | 0.0079 | 5.58E-68 |  |  | BMI | 0.1345 | 0.0283 | 1.93E-06 |
| AC123768.3.std | -0.0194 | 0.0444 | 0.6627 |  |  | AC123768.3.ter | -0.0398 | 0.1516 | 0.7931 |
| BMI_x_AC123768.3.std | 0.0031 | 0.0081 | 0.6996 |  |  | BMI_x_AC123768.3.ter | 0.0035 | 0.0274 | 0.8996 |
| **BMI by AC123768.3 Tertiles** | | | |  |  |  |  |  |  |
| *AC123768.3* < -0.75 | 0.1238 | 0.0380 | 1.12E-03 | 3872 |  |  |  |  |  |
| =-0.75 < *AC123768.3* < 0.75 | 0.1388 | 0.0083 | 2.06E-63 | 77819 |  |  |  |  |  |
| *AC123768.3* > 0.75 | 0.1307 | 0.0425 | 2.13E-03 | 3175 |  |  |  |  |  |
|  |  |  |  |  |  |  |  |  |  |
| ***ARHGAP11A*** | | | |  |  |  |  |  |  |
| **Expression Only** |  |  |  |  |  | **Expression Only** |  |  |  |
| BMI | 0.1380 | 0.0079 | 5.32E-68 |  |  | BMI | 0.1380 | 0.0079 | 5.29E-68 |
| ARHGAP11A.std | 0.0133 | 0.0074 | 0.0715 |  |  | ARHGAP11A.quar | 0.0153 | 0.0086 | 0.0749 |
| **Interaction Model** |  |  |  |  |  | **Interaction Model** |  |  |  |
| BMI | 0.1380 | 0.0079 | 5.37E-68 |  |  | BMI | 0.1421 | 0.0154 | 2.74E-20 |
| ARHGAP11A.std | 0.0067 | 0.0434 | 0.8771 |  |  | ARHGAP11A.quar | 0.0309 | 0.0506 | 0.5408 |
| BMI_x_ARHGAP11A.std | 0.0012 | 0.0078 | 0.8783 |  |  | BMI_x_ARHGAP11A.quar | -0.0029 | 0.0091 | 0.7538 |
| **BMI by ARHGAP11A Quartiles** | | | |  |  |  |  |  |  |
| *ARHGAP11A* < -1 | 0.1344 | 0.0206 | 6.47E-11 | 12796 |  |  |  |  |  |
| =-1 < *ARHGAP11A* < 0 | 0.1388 | 0.0135 | 5.89E-25 | 29278 |  |  |  |  |  |
| 0 < *ARHGAP11A* < 1.2 | 0.1466 | 0.0124 | 1.88E-32 | 35115 |  |  |  |  |  |
| *ARHGAP11A* > 1.2 | 0.1110 | 0.0263 | 2.44E-05 | 7677 |  |  |  |  |  |
|  |  |  |  |  |  |  |  |  |  |
| ***AVEN*** | | | |  |  |  |  |  |  |
| **Expression Only** |  |  |  |  |  | **Expression Only** |  |  |  |
| BMI | 0.1380 | 0.0079 | 5.57E-68 |  |  | BMI | 0.1379 | 0.0079 | 5.69E-68 |
| AVEN.std | 0.0005 | 0.0072 | 0.9421 |  |  | AVEN.ter | 0.0029 | 0.0108 | 0.7899 |
| **Interaction Model** |  |  |  |  |  | **Interaction Model** |  |  |  |
| BMI | 0.1383 | 0.0079 | 2.94E-68 |  |  | BMI | 0.1049 | 0.0179 | 4.72E-09 |
| AVEN.std | -0.0581 | 0.0419 | 0.1661 |  |  | AVEN.ter | -0.1255 | 0.0633 | 0.0473 |
| BMI_x_AVEN.std | 0.0107 | 0.0076 | 0.1561 |  |  | BMI_x_AVEN.ter | 0.0235 | 0.0114 | 0.0395 |
| **BMI by AVEN Tertiles** | | | |  |  |  |  |  |  |
| AVEN < -0.7 | 0.1143 | 0.0237 | 1.43E-06 | 9540 |  |  |  |  |  |
| =-0.7 < AVEN < 0.1 | 0.1234 | 0.0130 | 3.03E-21 | 30759 |  |  |  |  |  |
| AVEN > 0.1 | 0.1546 | 0.0110 | 9.78E-45 | 44567 |  |  |  |  |  |
|  |  |  |  |  |  |  |  |  |  |
| ***CHRNA7*** | | | |  |  |  |  |  |  |
| **Expression Only** |  |  |  |  |  | **Expression Only** |  |  |  |
| BMI | 0.1379 | 0.0079 | 5.94E-68 |  |  | BMI | 0.1379 | 0.0079 | 5.72E-68 |
| CHRNA7.std | -0.0139 | 0.0075 | 0.0628 |  |  | CHRNA7.ter | -0.0057 | 0.0106 | 0.5922 |
| **Interaction Model** |  |  |  |  |  | **Interaction Model** |  |  |  |
| BMI | 0.1379 | 0.0079 | 6.16E-68 |  |  | BMI | 0.1586 | 0.0128 | 2.55E-35 |
| CHRNA7.std | 0.0297 | 0.0441 | 0.5009 |  |  | CHRNA7.ter | 0.1221 | 0.0629 | 0.0522 |
| BMI_x_CHRNA7.std | -0.0080 | 0.0080 | 0.3162 |  |  | BMI_x_CHRNA7.ter | -0.0234 | 0.0114 | 0.0392 |
| **BMI by CHRNA7 Tertiles** | | | |  |  |  |  |  |  |
| *CHRNA7* < -0.5 | 0.1680 | 0.0145 | 4.73E-31 | 25447 |  |  |  |  |  |
| =-0.5 < *CHRNA7* < 0.5 | 0.1259 | 0.0110 | 4.12E-30 | 43557 |  |  |  |  |  |
| *CHRNA7* > 0.5 | 0.1272 | 0.0185 | 5.83E-12 | 15862 |  |  |  |  |  |
|  |  |  |  |  |  |  |  |  |  |
| ***FMN1*** | | | |  |  |  |  |  |  |
| **Expression Only** |  |  |  |  |  | **Expression Only** |  |  |  |
| BMI | 0.1379 | 0.0079 | 6.52E-68 |  |  | BMI | 0.1379 | 0.0079 | 6.10E-68 |
| FMN1.std | -0.0076 | 0.0073 | 0.2991 |  |  | FMN1.ter | -0.0047 | 0.0104 | 0.6528 |
| **Interaction Model** |  |  |  |  |  | **Interaction Model** |  |  |  |
| BMI | 0.1379 | 0.0079 | 7.85E-68 |  |  | BMI | 0.1424 | 0.0152 | 9.73E-21 |
| FMN1.std | -0.0038 | 0.0427 | 0.9287 |  |  | FMN1.ter | 0.0161 | 0.0614 | 0.7930 |
| BMI_x_FMN1.std | -0.0007 | 0.0077 | 0.9292 |  |  | BMI_x_FMN1.ter | -0.0038 | 0.0111 | 0.7310 |
| **BMI by FMN1 Tertiles** | | | |  |  |  |  |  |  |
| *FMN1* < -1 | 0.1300 | 0.0190 | 7.33E-12 | 14482 |  |  |  |  |  |
| =-1 < *FMN1* < 0.35 | 0.1471 | 0.0115 | 2.12E-37 | 40105 |  |  |  |  |  |
| *FMN1* > 0.35 | 0.1303 | 0.0134 | 2.49E-22 | 30279 |  |  |  |  |  |
|  |  |  |  |  |  |  |  |  |  |
| ***RYR3*** | | | |  |  |  |  |  |  |
| **Expression Only** |  |  |  |  |  | **Expression Only** |  |  |  |
| BMI | 0.1379 | 0.0079 | 5.74E-68 |  |  | BMI | 0.1379 | 0.0079 | 5.74E-68 |
| RYR3.std | 0.0106 | 0.0074 | 0.1488 |  |  | RYR3.quar | 0.0093 | 0.0089 | 0.2981 |
| **Interaction Model** |  |  |  |  |  | **Interaction Model** |  |  |  |
| BMI | 0.1378 | 0.0079 | 7.97E-68 |  |  | BMI | 0.1443 | 0.0144 | 8.76E-24 |
| RYR3.std | 0.0391 | 0.0436 | 0.3703 |  |  | RYR3.quar | 0.0373 | 0.0530 | 0.4821 |
| BMI_x_RYR3.std | -0.0052 | 0.0079 | 0.5082 |  |  | BMI_x_RYR3.quar | -0.0051 | 0.0096 | 0.5926 |
| **BMI by RYR3 Quartiles** | | | |  |  |  |  |  |  |
| *RYR3* < -0.9 | 0.1378 | 0.0190 | 3.68E-13 | 15299 |  |  |  |  |  |
| =-0.9 < *RYR3* < 0.25 | 0.1472 | 0.0117 | 3.90E-36 | 38328 |  |  |  |  |  |
| 0.25 < *RYR3* < 1.25 | 0.1209 | 0.0144 | 3.51E-17 | 25983 |  |  |  |  |  |
| *RYR3* > 1.25 | 0.1524 | 0.0323 | 2.39E-06 | 5256 |  |  |  |  |  |

The gene expression was standardized to have a mean of 0 and variance of 1

**Supplementary methods**

For methods description, we adopt the following notation: E = BMI, G = SNP, D = outcome (CRC), and C = a set of adjustment covariables. We tested multiplicative interactions by fitting a conventional logistic regression model in the form $logit\left( \Pr\left( D=1 | G \right) \right)=\beta_{0}+\beta_{G}G+\beta_{E}E+ \beta_{GxE}GxE+\beta_{C}C$, where assessing $H0:\beta_{GxE}=0$ tests potential departures from multiplicative scale interactions between E and G on D.

We also implemented the EDGE two-step method that prioritizes GxE interactions tests (step 2) based on ranks from a filtering statistic (step 1) under a weighted hypothesis testing framework^1^ .The Step 1 screen is based on a 2DF test of the CRC vs. G (“D|G”) and BMI vs. G (“E|G”) associations. P-values from this screening test are computed for each SNP (G) and ranked from smallest (most significant) to largest. The Step-2 test is based on the standard 1DF GxE test. The 2DF screening test, as well as the component D|G and E|G statistics, are independent of the Step-2 GxE test, a key requirement to preserve the overall family-wise error rate for the two-step scanning procedure ^1-3^. The original approach uses step 1 ranks to prioritize and partition SNPs into exponentially larger bins of fixed sizes and increasingly more stringent step 2 significance thresholds ^4^. However, when analyzing imputed SNPs, highly correlated markers from the same loci populate the top bins, diminishing statistical power. Thus, we modified the original weighted hypothesis testing framework to accommodate bins of varying sizes while properly controlling for type I error ^5^. Specifically, SNPs were partitioned into bins based on step 1 *p*-value thresholds in expectation, which were calculated using the original predetermined bin sizes (initial bin size of 5 and overall alpha = 0.05) and assumed uniform distribution of 1 million independent tests. For step 2 GxE testing, we accounted for the influx of correlated markers into each bin by correcting for the effective number of tests, estimated using PCA performed on bin specific genotype correlation matrices ^6^. This modification alleviates multiple testing burden and improves statistical power, while maintaining an overall type I error rate of 0.05.

Joint tests of association can improve power to detect disease susceptibility loci in a wider variety of circumstances by accounting for GxE interactions, e.g., in situations where susceptibility loci affect only individuals with certain environmental exposure profiles. One possibility is the 2DF joint test, which tests the null hypothesis $H0=\beta_{G}=\beta_{GxE}=0$ based on the standard logistic regression model^7^ By reparametrizing the interaction model, we can extend the 2DF testing framework to include E|G associations and perform a 3DF test in the form $H0=\beta_{G}=\beta_{GxE}=\gamma_{G}=0$, where $\gamma_{G}$ represents the association between G and E in the combined case-control sample ^3,8^. We utilized the 3DF joint test in our analysis as it provides power than the 2DF test for most underlying models of GxE interaction.^7^

No single GxE test is universally most powerful and with that in mind, we applied three different testing procedures to maximize the chance of discovering novel loci related to CRC. These include the standard one-degree-of-freedom (1df) test of GxE interaction, the 2-step EDGE procedure, and the 3df joint test.

*Functional follow-up*

We created regional plots for statistically significant findings to more deeply examine magnitudes of association, the extent of association signal due to LD, and chromosomal position of findings relative to genes in the given region. Plots were generated using the command line version (Standalone) of LocusZoom v1.3 ^9^. Measures of LD were estimated using European populations from the 1000 Genomes Project. The putative functional role of these SNPs and those in LD (r2 > 0.2) at 500kb flanking regions were investigated relative to their potential contribution to regulate gene expression by their physical location in regions of chromatin accessibility or histone modifications (variant enhancer loci). Regions containing active enhancer elements in tissue from healthy colon and from colon tumor tissue samples were obtained from previously analyzed ATAC-seq, DNase Hypersensitivity (DHS)-seq, and H3K27ac histone ChIP-seq datasets. Genes where expression in colon tissue samples was regulated by functional SNPs (P < 5x10-4) were identified using different databases: the colon transverse tissue samples from GTEx v8 dataset, and the colon transcriptome explorer (CoTrEx 2.0; https://barcuvaseq.org/cotrex, accessed January 2023) of the University of Barcelona and University of Virginia genotyping and RNA sequencing (BarcUVA-Seq) project dataset ^10^, which is comprised of 445 epithelium-enriched healthy colon biopsies from ascending, transverse, and descending colon.

We queried overlaps between our findings and regions containing active enhancer elements in tissue from healthy and tumor colon samples in addition to CRC cell lines, obtained from previously analyzed ATAC-seq, DNase Hypersensitivity (DHS)-seq, and H3K27ac histone ChIP-seq dataset ^11^. We extended this analysis to include additional tissue types by incorporating regulatory annotations of histone modifications from 10 groups of tissues, obtained from several resources ^12,13^ and compiled by Finucane et al. ^14^. Furthermore, we queried lead and LD SNPs against functional annotation databases from ENSEMBL using the Variant Effect Predictor tool ^15^.

**References**

1. Gauderman, W.J., Zhang, P., Morrison, J.L., and Lewinger, J.P. (2013). Finding novel genes by testing G × E interactions in a genome-wide association study. Genet Epidemiol *37*, 603-613. 10.1002/gepi.21748.

2. Kooperberg, C., and Leblanc, M. (2008). Increasing the power of identifying gene x gene interactions in genome-wide association studies. Genetic epidemiology *32*, 255-263. 10.1002/gepi.20300.

3. Murcray, C.E., Lewinger, J.P., and Gauderman, W.J. (2009). Gene-environment interaction in genome-wide association studies. Am J Epidemiol *169*, 219-226. 10.1093/aje/kwn353.

4. Ionita-Laza, I., McQueen, M.B., Laird, N.M., and Lange, C. (2007). Genomewide weighted hypothesis testing in family-based association studies, with an application to a 100K scan. Am J Hum Genet *81*, 607-614. 10.1086/519748.

5. Kawaguchi, E.S., Kim, A.E., Lewinger, J.P., and Gauderman, W.J. (2022). Improved two-step testing of genome-wide gene-environment interactions. bioRxiv, 2022.2006.2014.496154. 10.1101/2022.06.14.496154.

6. Gao, X., Starmer, J., and Martin, E.R. (2008). A multiple testing correction method for genetic association studies using correlated single nucleotide polymorphisms. Genet Epidemiol *32*, 361-369. 10.1002/gepi.20310.

7. Kraft, P., Yen, Y.C., Stram, D.O., Morrison, J., and Gauderman, W.J. (2007). Exploiting gene-environment interaction to detect genetic associations. Hum Hered *63*, 111-119. 10.1159/000099183.

8. Gauderman, W.J., Kim, A., Conti, D.V., Morrison, J., Thomas, D.C., Vora, H., and Lewinger, J.P. (2019). A Unified Model for the Analysis of Gene-Environment Interaction. American journal of epidemiology *188*, 760-767. 10.1093/aje/kwy278.

9. Pruim, R.J., Welch, R.P., Sanna, S., Teslovich, T.M., Chines, P.S., Gliedt, T.P., Boehnke, M., Abecasis, G.R., and Willer, C.J. (2010). LocusZoom: regional visualization of genome-wide association scan results. Bioinformatics *26*, 2336-2337. 10.1093/bioinformatics/btq419.

10. Díez-Obrero, V., Dampier, C.H., Moratalla-Navarro, F., Devall, M., Plummer, S.J., Díez-Villanueva, A., Peters, U., Bien, S., Huyghe, J.R., Kundaje, A., et al. (2021). Genetic Effects on Transcriptome Profiles in Colon Epithelium Provide Functional Insights for Genetic Risk Loci. Cell Mol Gastroenterol Hepatol *12*, 181-197. 10.1016/j.jcmgh.2021.02.003.

11. Cohen, A.J., Saiakhova, A., Corradin, O., Luppino, J.M., Lovrenert, K., Bartels, C.F., Morrow, J.J., Mack, S.C., Dhillon, G., Beard, L., et al. (2017). Hotspots of aberrant enhancer activity punctuate the colorectal cancer epigenome. Nat Commun *8*, 14400. 10.1038/ncomms14400.

12. Trynka, G., Sandor, C., Han, B., Xu, H., Stranger, B.E., Liu, X.S., and Raychaudhuri, S. (2013). Chromatin marks identify critical cell types for fine mapping complex trait variants. Nat Genet *45*, 124-130. 10.1038/ng.2504.

13. Hnisz, D., Abraham, B.J., Lee, T.I., Lau, A., Saint-André, V., Sigova, A.A., Hoke, H.A., and Young, R.A. (2013). Super-enhancers in the control of cell identity and disease. Cell *155*, 934-947. 10.1016/j.cell.2013.09.053.

14. Finucane, H.K., Bulik-Sullivan, B., Gusev, A., Trynka, G., Reshef, Y., Loh, P.R., Anttila, V., Xu, H., Zang, C., Farh, K., et al. (2015). Partitioning heritability by functional annotation using genome-wide association summary statistics. Nat Genet *47*, 1228-1235. 10.1038/ng.3404.

15. McLaren, W., Gil, L., Hunt, S.E., Riat, H.S., Ritchie, G.R., Thormann, A., Flicek, P., and Cunningham, F. (2016). The Ensembl Variant Effect Predictor. Genome Biol *17*, 122. 10.1186/s13059-016-0974-4.

**Funding/acknowledgements**

Elom K. Aglago’s associateship was supported by a Cancer Research UK (CRUK) grant awarded to Kostas Tsilidis (PPRCPJT\100005). Genetics and Epidemiology of Colorectal Cancer Consortium (GECCO): National Cancer Institute, National Institutes of Health, U.S. Department of Health and Human Services (U01 CA137088, R01 CA059045, U01 CA164930, R01201407). Genotyping/Sequencing services were provided by the Center for Inherited Disease Research (CIDR) contract number HHSN268201700006I and HHSN268201200008I. This research was funded in part through the NIH/NCI Cancer Center Support Grant P30 CA015704. Scientific Computing Infrastructure at Fred Hutch funded by ORIP grant S10OD028685.

The ATBC Study is supported by the Intramural Research Program of the U.S. National Cancer Institute, National Institutes of Health, Department of Health and Human Services.

CLUE II funding was from the National Cancer Institute (U01 CA86308, Early Detection Research Network; P30 CA006973), National Institute on Aging (U01 AG18033), and the American Institute for Cancer Research. The content of this publication does not necessarily reflect the views or policies of the Department of Health and Human Services, nor does mention of trade names, commercial products, or organizations imply endorsement by the US government.

Maryland Cancer Registry (MCR)

Cancer data was provided by the Maryland Cancer Registry, Center for Cancer Prevention and Control, Maryland Department of Health, with funding from the State of Maryland and the Maryland Cigarette Restitution Fund. The collection and availability of cancer registry data is also supported by the Cooperative Agreement NU58DP006333, funded by the Centers for Disease Control and Prevention. Its contents are solely the responsibility of the authors and do not necessarily represent the official views of the Centers for Disease Control and Prevention or the Department of Health and Human Services.

The Colon Cancer Family Registry (CCFR, www.coloncfr.org) is supported in part by funding from the National Cancer Institute (NCI), National Institutes of Health (NIH) (award U01 CA167551). Support for case ascertainment was provided in part from the Surveillance, Epidemiology, and End Results (SEER) Program and the following U.S. state cancer registries: AZ, CO, MN, NC, NH; and by the Victoria Cancer Registry (Australia) and Ontario Cancer Registry (Canada). The CCFR Set-1 (Illumina 1M/1M-Duo) and Set-2 (Illumina Omni1-Quad) scans were supported by NIH awards U01 CA122839 and R01 CA143247 (to GC). The CCFR Set-3 (Affymetrix Axiom CORECT Set array) was supported by NIH award U19 CA148107 and R01 CA81488 (to SBG). The CCFR Set-4 (Illumina OncoArray 600K SNP array) was supported by NIH award U19 CA148107 (to SBG) and by the Center for Inherited Disease Research (CIDR), which is funded by the NIH to the Johns Hopkins University, contract number HHSN268201200008I. Additional funding for the OFCCR/ARCTIC was through award GL201-043 from the Ontario Research Fund (to BWZ), award 112746 from the Canadian Institutes of Health Research (to TJH), through a Cancer Risk Evaluation (CaRE) Program grant from the Canadian Cancer Society (to SG), and through generous support from the Ontario Ministry of Research and Innovation. The SFCCR Illumina HumanCytoSNP array was supported in part through NCI/NIH awards U01/U24 CA074794 and R01 CA076366 (to PAN). The content of this manuscript does not necessarily reflect the views or policies of the NCI, NIH or any of the collaborating centers in the Colon Cancer Family Registry (CCFR), nor does mention of trade names, commercial products, or organizations imply endorsement by the US Government, any cancer registry, or the CCFR.

Colorectal Cancer Transdisciplinary (CORECT) Study: The CORECT Study was supported by the National Cancer Institute, National Institutes of Health (NCI/NIH), U.S. Department of Health and Human Services (grant numbers U19 CA148107, R01 CA81488, P30 CA014089, R01 CA197350; P01 CA196569; R01 CA201407) and National Institutes of Environmental Health Sciences, National Institutes of Health (grant number T32 ES013678).

CORSA: The CORSA study was funded by Austrian Research Funding Agency (FFG) BRIDGE (grant 829675, to Andrea Gsur), the “Herzfelder’sche Familienstiftung” (grant to Andrea Gsur) and was supported by COST Action BM1206.

CPS-II: The American Cancer Society funds the creation, maintenance, and updating of the Cancer Prevention Study-II (CPS-II) cohort. This study was conducted with Institutional Review Board approval.

CRCGEN: Colorectal Cancer Genetics & Genomics, Spanish study was supported by Instituto de Salud Carlos III, co-funded by FEDER funds –a way to build Europe– (grants PI14-613 and PI09-1286), Agency for Management of University and Research Grants (AGAUR) of the Catalan Government (grant 2017SGR723), and Junta de Castilla y León (grant LE22A10-2). Sample collection of this work was supported by the Xarxa de Bancs de Tumors de Catalunya sponsored by Pla Director d’Oncología de Catalunya (XBTC), Plataforma Biobancos PT13/0010/0013 and ICOBIOBANC, sponsored by the Catalan Institute of Oncology.

Czech Republic CCS: This work was supported by the Grant Agency of the Czech Republic (18-09709S, 20-03997S), by the Grant Agency of the Ministry of Health of the Czech Republic (grants AZV NV18/03/00199 and AZV NV19-09-00237), and Charles University grants Unce/Med/006 and Progress Q28/LF1.

DACHS: This work was supported by the German Research Council (BR 1704/6-1, BR 1704/6-3, BR 1704/6-4, CH 117/1-1, HO 5117/2-1, HE 5998/2-1, KL 2354/3-1, RO 2270/8-1 and BR 1704/17-1), the Interdisciplinary Research Program of the National Center for Tumor Diseases (NCT), Germany, and the German Federal Ministry of Education and Research (01KH0404, 01ER0814, 01ER0815, 01ER1505A and 01ER1505B).

DALS: National Institutes of Health (R01 CA48998 to M. L. Slattery).

EDRN: This work is funded and supported by the NCI, EDRN Grant (U01 CA 84968-06).

EPIC: The coordination of EPIC is financially supported by International Agency for Research on Cancer (IARC) and also by the Department of Epidemiology and Biostatistics, School of Public Health, Imperial College London which has additional infrastructure support provided by the NIHR Imperial Biomedical Research Centre (BRC). The national cohorts are supported by: Danish Cancer Society (Denmark); Ligue Contre le Cancer, Institut Gustave Roussy, Mutuelle Générale de l’Education Nationale, Institut National de la Santé et de la Recherche Médicale (INSERM) (France); German Cancer Aid, German Cancer Research Center (DKFZ), German Institute of Human Nutrition Potsdam- Rehbruecke (DIfE), Federal Ministry of Education and Research (BMBF) (Germany); Associazione Italiana per la Ricerca sul Cancro-AIRC-Italy, Compagnia di SanPaolo and National Research Council (Italy); Dutch Ministry of Public Health, Welfare and Sports (VWS), Netherlands Cancer Registry (NKR), LK Research Funds, Dutch Prevention Funds, Dutch ZON (Zorg Onderzoek Nederland), World Cancer Research Fund (WCRF), Statistics Netherlands (The Netherlands); Health Research Fund (FIS) - Instituto de Salud Carlos III (ISCIII), Regional Governments of Andalucía, Asturias, Basque Country, Murcia and Navarra, and the Catalan Institute of Oncology - ICO (Spain); Swedish Cancer Society, Swedish Research Council and County Councils of Skåne and Västerbotten (Sweden); Cancer Research UK (14136 to EPIC-Norfolk; C8221/A29017 to EPIC-Oxford), Medical Research Council (1000143 to EPIC-Norfolk; MR/M012190/1 to EPIC-Oxford). (United Kingdom).

ESTHER/VERDI. This work was supported by grants from the Baden-Württemberg Ministry of Science, Research and Arts and the German Cancer Aid.

Harvard cohorts: HPFS is supported by the National Institutes of Health (P01 CA055075, UM1 CA167552, U01 CA167552, R01 CA137178, R01 CA151993, and R35 CA197735), NHS by the National Institutes of Health (P01 CA087969, UM1 CA186107, R01 CA137178, R01 CA151993, and R35 CA197735), and PHS by the National Institutes of Health (R01 CA042182).

Hawaii Adenoma Study: NCI grants R01 CA72520.

Kentucky: This work was supported by the following grant support: Clinical Investigator Award from Damon Runyon Cancer Research Foundation (CI-8); NCI R01CA136726.

LCCS: The Leeds Colorectal Cancer Study was funded by the Food Standards Agency and Cancer Research UK Programme Award (C588/A19167).

MCCS cohort recruitment was funded by VicHealth and Cancer Council Victoria. The MCCS was further supported by Australian NHMRC grants 509348, 209057, 251553 and 504711 and by infrastructure provided by Cancer Council Victoria. Cases and their vital status were ascertained through the Victorian Cancer Registry (VCR) and the Australian Institute of Health and Welfare (AIHW), including the National Death Index and the Australian Cancer Database.

MEC: National Institutes of Health (R37 CA54281, P01 CA033619, and R01 CA063464).

MECC: This work was supported by the National Institutes of Health, U.S. Department of Health and Human Services (R01 CA81488, R01 CA197350).

NCCCS I & II: We acknowledge funding support for this project from the National Institutes of Health, R01 CA66635 and P30 DK034987.

NFCCR: This work was supported by an Interdisciplinary Health Research Team award from the Canadian Institutes of Health Research (CRT 43821); the National Institutes of Health, U.S. Department of Health and Human Serivces (U01 CA74783); and National Cancer Institute of Canada grants (18223 and 18226). The authors wish to acknowledge the contribution of Alexandre Belisle and the genotyping team of the McGill University and Génome Québec Innovation Centre, Montréal, Canada, for genotyping the Sequenom panel in the NFCCR samples. Funding was provided to Michael O. Woods by the Canadian Cancer Society Research Institute.

PLCO: Intramural Research Program of the Division of Cancer Epidemiology and Genetics and supported by contracts from the Division of Cancer Prevention, National Cancer Institute, NIH, DHHS. Funding was provided by National Institutes of Health (NIH), Genes, Environment and Health Initiative (GEI) Z01 CP 010200, NIH U01 HG004446, and NIH GEI U01 HG 004438.

SELECT: Research reported in this publication was supported in part by the National Cancer Institute of the National Institutes of Health under Award Numbers U10 CA37429 (CD Blanke), and UM1 CA182883 (CM Tangen/IM Thompson). The content is solely the responsibility of the authors and does not necessarily represent the official views of the National Institutes of Health.

SMS: This work was supported by the National Cancer Institute (grant P01 CA074184 to J.D.P. and P.A.N., grants R01 CA097325, R03 CA153323, and K05 CA152715 to P.A.N., and the National Center for Advancing Translational Sciences at the National Institutes of Health (grant KL2 TR000421 to A.N.B.-H.)

REACH: This work was supported by the National Cancer Institute (grant P01 CA074184 to J.D.P. and P.A.N., grants R01 CA097325, R03 CA153323, and K05 CA152715 to P.A.N., and the National Center for Advancing Translational Sciences at the National Institutes of Health (grant KL2 TR000421 to A.N.B.-H.)

UK Biobank: This research has been conducted using the UK Biobank Resource under Application Number 8614

VITAL: National Institutes of Health (K05 CA154337).

WHI: The WHI program is funded by the National Heart, Lung, and Blood Institute, National Institutes of Health, U.S. Department of Health and Human Services through contracts HHSN268201100046C, HHSN268201100001C, HHSN268201100002C, HHSN268201100003C, HHSN268201100004C, and HHSN271201100004C.

Acknowledgements:

CCFR: The Colon CFR graciously thanks the generous contributions of their study participants, dedication of study staff, and the financial support from the U.S. National Cancer Institute, without which this important registry would not exist. The authors would like to thank the study participants and staff of the Seattle Colon Cancer Family Registry and the Hormones and Colon Cancer study (CORE Studies).

CLUE II: We thank the participants of Clue II and appreciate the continued efforts of the staff at the Johns Hopkins George W. Comstock Center for Public Health Research and Prevention in the conduct of the Clue II Cohort Study.

CORSA: We kindly thank all individuals who agreed to participate in the CORSA study. Furthermore, we thank all cooperating physicians and students and the Biobank Graz of the Medical University of Graz.

CPS-II: The authors thank the CPS-II participants and Study Management Group for their invaluable contributions to this research. The authors would also like to acknowledge the contribution to this study from central cancer registries supported through the Centers for Disease Control and Prevention National Program of Cancer Registries, and cancer registries supported by the National Cancer Institute Surveillance Epidemiology and End Results program.

Czech Republic CCS: We are thankful to all clinicians in major hospitals in the Czech Republic, without whom the study would not be practicable. We are also sincerely grateful to all patients participating in this study.

DACHS: We thank all participants and cooperating clinicians, and everyone who provided excellent technical assistance.

EDRN: We acknowledge all contributors to the development of the resource at University of Pittsburgh School of Medicine, Department of Gastroenterology, Department of Pathology, Hepatology and Nutrition and Biomedical Informatics.

EPIC: Where authors are identified as personnel of the International Agency for Research on Cancer/World Health Organization, the authors alone are responsible for the views expressed in this article and they do not necessarily represent the decisions, policy or views of the International Agency for Research on Cancer/World Health Organization.

Harvard cohorts (HPFS, NHS, PHS): The study protocol was approved by the institutional review boards of the Brigham and Women’s Hospital and Harvard T.H. Chan School of Public Health, and those of participating registries as required. We acknowledge Channing Division of Network Medicine, Department of Medicine, Brigham and Women's Hospital as home of the NHS. The authors would like to acknowledge the contribution to this study from central cancer registries supported through the Centers for Disease Control and Prevention’s National Program of Cancer Registries (NPCR) and/or the National Cancer Institute’s Surveillance, Epidemiology, and End Results (SEER) Program. Central registries may also be supported by state agencies, universities, and cancer centers. Participating central cancer registries include the following: Alabama, Alaska, Arizona, Arkansas, California, Colorado, Connecticut, Delaware, Florida, Georgia, Hawaii, Idaho, Indiana, Iowa, Kentucky, Louisiana, Massachusetts, Maine, Maryland, Michigan, Mississippi, Montana, Nebraska, Nevada, New Hampshire, New Jersey, New Mexico, New York, North Carolina, North Dakota, Ohio, Oklahoma, Oregon, Pennsylvania, Puerto Rico, Rhode Island, Seattle SEER Registry, South Carolina, Tennessee, Texas, Utah, Virginia, West Virginia, Wyoming. The authors assume full responsibility for analyses and interpretation of these data.

Kentucky: We would like to acknowledge the staff at the Kentucky Cancer Registry.

LCCS: We acknowledge the contributions of Jennifer Barrett, Robin Waxman, Gillian Smith and Emma Northwood in conducting this study.

NCCCS I & II: We would like to thank the study participants, and the NC Colorectal Cancer Study staff.

PLCO: The authors thank the PLCO Cancer Screening Trial screening center investigators and the staff from Information Management Services Inc and Westat Inc. Most importantly, we thank the study participants for their contributions that made this study possible.

Cancer incidence data have been provided by the District of Columbia Cancer Registry, Georgia Cancer Registry, Hawaii Cancer Registry, Minnesota Cancer Surveillance System, Missouri Cancer Registry, Nevada Central Cancer Registry, Pennsylvania Cancer Registry, Texas Cancer Registry, Virginia Cancer Registry, and Wisconsin Cancer Reporting System. All are supported in part by funds from the Center for Disease Control and Prevention, National Program for Central Registries, local states or by the National Cancer Institute, Surveillance, Epidemiology, and End Results program. The results reported here and the conclusions derived are the sole responsibility of the authors.

SELECT: We thank the research and clinical staff at the sites that participated on SELECT study, without whom the trial would not have been successful. We are also grateful to the 35,533 dedicated men who participated in SELECT.

WHI: The authors thank the WHI investigators and staff for their dedication, and the study participants for making the program possible. A full listing of WHI investigators can be found at: http://www.whi.org/researchers/Documents%20%20Write%20a%20Paper/WHI%20Investigator%20Short%20List.pdf
